# TNFα sensitizes hepatocytes to FasL-induced apoptosis by NFκB-mediated Fas upregulation

**DOI:** 10.1038/s41419-018-0935-9

**Published:** 2018-09-05

**Authors:** Laura Faletti, Lukas Peintner, Simon Neumann, Sandra Sandler, Thomas Grabinger, Sabine Mac Nelly, Irmgard Merfort, Chun-Hao Huang, Darjus Tschaharganeh, Tae-Won Kang, Florian Heinzmann, Luana D’Artista, Ulrich Maurer, Thomas Brunner, Scott Lowe, Lars Zender, Christoph Borner

**Affiliations:** 1 Institute of Molecular Medicine and Cell Research, Faculty of Medicine, Albert Ludwigs University Freiburg, Stefan Meier Strasse 17, D-79104 Freiburg, Germany; 2 Faculty of Biology, Albert Ludwigs University Freiburg, Schänzlestrasse 1, D-79104 Freiburg, Germany; 3 Spemann Graduate School of Biology and Medicine (SGBM), Albert Ludwigs University Freiburg, Albertstrasse 19a, D-79104 Freiburg, Germany; 4 Biochemical Pharmacology, Department of Biology, University of Konstanz, Universitätsstrasse 10, D-78457 Konstanz, Germany; 5 Department of Internal Medicine, University Medical Center Freiburg, Hugstetter Strasse 55, D-79106 Freiburg, Germany; 6 Department of Pharmacology, Biology and Biotechnology, Albert Ludwigs University Freiburg, Stefan Meier Strasse 19, D-79104 Freiburg, Germany; 7 Department of Cancer Biology and Genetics, Memorial Sloan Kettering Cancer Center, New York, New York 10065 USA; 8 Department Internal Medicine VIII, University Medical Center Tübingen, Otfried Müller Strasse 14, D-72076 Tübingen, Germany; 9 Department of Physiology I, Institute of Physiology, Eberhard Karls University Tübingen, Wilhalmstrasse 56, D-72076 Tübingen, Germany; 10 Translational Gastrointenstinal Oncology Group, German Consortium for Translational Cancer Research (DKTK), German Cancer Research Center (DKFZ), Im Neuenheimer Feld 224, D-69120 Heidelberg, Germany; 11 BIOSS, Centre for Biological Signalling Studies, Schänzlestrasse 14, D-79104 Freiburg, Germany; 12 Present Address: Institute of Physiology, University of Zürich, Winterthurerstrasse 190, CH-8057 Zürich, Switzerland; 13 Present Address: Department of Molecular and Cell Biology, University of California, Berkeley, CA 94720 USA; 14 Present Address: Helmholtz Group Cell Plasticity and Epigenetic Remodeling, German Cancer Research Center (DKFZ) & Institute of Pathology, University Hospital, Im Neuenheimer Feld 224, D-69120 Heidelberg, Germany

## Abstract

Although it is well established that TNFα contributes to hepatitis, liver failure and associated hepatocarcinogenesis via the regulation of inflammation, its pro-apoptotic role in the liver has remained enigmatic. On its own, TNFα is unable to trigger apoptosis. However, when combined with the transcriptional inhibitor GaLN, it can cause hepatocyte apoptosis and liver failure in mice. Moreover, along with others, we have shown that TNFα is capable of sensitizing cells to FasL- or drug-induced cell death via c-Jun N-terminal kinase (JNK) activation and phosphorylation/activation of the BH3-only protein Bim. In this context, TNFα could exacerbate hepatocyte cell death during simultaneous inflammatory and T-cell-mediated immune responses in the liver. Here we show that TNFα sensitizes primary hepatocytes, established hepatocyte cell lines and mouse embryo fibroblasts to FasL-induced apoptosis by the transcriptional induction and higher surface expression of Fas via the NFκB pathway. Genetic deletion, diminished expression or dominant-negative inhibition of the NFκB subunit p65 resulted in lower Fas expression and inhibited TNFα-induced Fas upregulation and sensitization to FasL-induced cell death. By hydrodynamic injection of p65 shRNA into the tail vein of mice, we confirm that Fas upregulation by TNFα is also NFκB-mediated in the liver. In conclusion, TNFα sensitization of FasL-induced apoptosis in the liver proceeds via two parallel signaling pathways, activation of JNK and Bim phosphorylation and NFκB-mediated Fas upregulation.

## Introduction

The death receptor Fas (CD95/APO-1) plays a central role in maintaining liver homeostasis by contributing to the removal of senescent, virus infected and cancer cells. Engagement of Fas by its cognate ligand (FasL) triggers a caspase-8/-3-dependent signaling cascade resulting in apoptotic cell death. In particular, hepatocytes constitutively express Fas^1^ and are susceptible to Fas-mediated apoptosis in vitro^2^. Moreover, mice injected with anti-Fas agonistic antibodies exhibit massive hepatocyte apoptosis and die of fulminant liver failure within a short time period^3,4^. Fas-mediated hepatocyte apoptosis is a common pathological feature of several human liver diseases^5–11^.

Activation of tumor necrosis factor receptor 1 (TNFR1), unlike Fas, does not primarily lead to cell death in most cell types^12^. Upon binding of TNFα to TNFR1, complex 1 is assembled leading to nuclear factor 'kappa-light-chain-enhancer' of activated B-cells (NFκB) activation, which induces a transcriptional program regulating inflammation, survival and proliferation. However, under specific conditions, engagement of TNFR1 leads to the formation complex 2 or the necrosomal complex, which foster cell death by apoptosis or necroptosis, respectively^13^. The transcription factor NFκB plays a crucial role in maintaining the balance between survival and death because of its ability to induce various anti-apoptotic and inflammatory proteins^14–17^. Therefore, an acute treatment of mice with TNFα only provokes hepatocyte cell death and liver injury when combined with transcriptional arrest such as the co-treatment with actinomycin D (ActD) or d-galactosamine (GaLN)^18^. The administration of lipopolysaccharide (LPS) (which induces TNFα production) to GaLN-sensitized mice has therefore been widely used as an experimental model for endotoxic shock^19–21^. In this model, liver injury indeed depends on the action of TNFα.

The initial wave of hepatotoxicity is often insufficient to cause fatal liver injury while a second step involving activation of the immune system eventually exacerbates tissue damage causing liver failure. TNFα, which is mainly produced by activated macrophages during inflammation, has been implicated as an important pathogenic mediator during liver diseases. Indeed, increased levels of TNFα have been found in the serum and livers of patients with chronic and acute hepatitis^22–24^. Moreover, Minagawa and colleagues unraveled a cooperative contribution of Fas and TNFR1 to chronic alcohol-induced liver injury^25^. This is in agreement with reports showing that fulminant liver injury induced by the injection of agonistic anti-Fas antibody is suppressed in TNFR1 defective mice^26^ and basal resistance of lung fibroblasts to Fas-induced apoptosis could be overcome by sensitization with TNFα^27^. Consistent with these findings, we previously reported that TNFα can enhance FasL-mediated cytotoxicity in isolated primary mouse hepatocytes via a JNK/Bim-dependent pathway^28^. However, c-Jun N-terminal kinase (JNK) inhibition or Bim deletion did not fully rescue the cells from TNF-induced apoptosis sensitization indicating there must be another crosstalk between TNFα- and FasL-induced signaling, which increases hepatocyte cell death and contributes to liver diseases.

Previous studies revealed that TNFα is able to upregulate Fas in mouse embryonic fibroblasts^29^, acute myeloid leukemia cell lines^30^ and neuroblastoma cells^31^. A binding site for the transcription factor NFκB was described in the Fas promoter, which regulates activation-dependent Fas expression in lymphocytes^32^. NFκB was also found to mediate transcriptional activation of Fas in hepatocytes during adenoviral hepatitis^33^ although increased Fas surface expression and higher sensitivity to FasL-induced apoptosis were not examined.

In the present study, we found that in addition to activating the JNK/Bim pathway, TNFα sensitizes to FasL-induced cell death of hepatocytes by upregulating Fas surface expression through an NFκB-mediated transcriptional induction of the Fas gene. This mechanism is also observed in the liver in vivo after treating mice with LPS or TNFα indicating that TNFα does not only engage NFκB to induced inflammatory and survival processes in the liver, but also to sensitize the organ to potential damage by FasL.

## Results

### TNFα enhances FasL-mediated apoptosis in hepatocytes

**Fig. 1:**
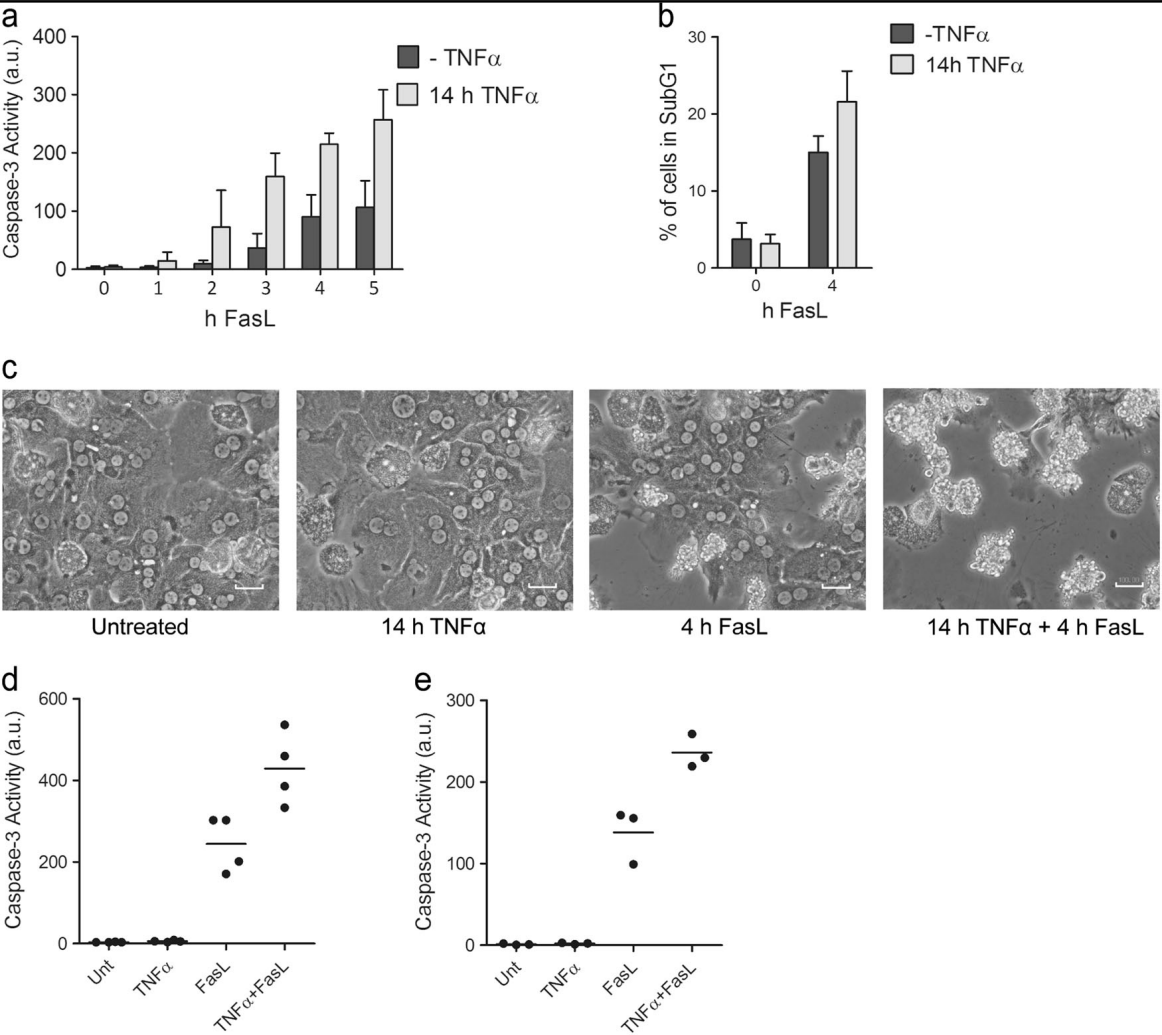
TNFα pretreatment enhances FasL-induced apoptosis. **a** Caspase-3/-7 activity in total cell lysates of primary mouse hepatocytes, treated with FasL alone for 0–5 h (-TNFα) or pretreated with TNFα for 14 h, followed by treatment with FasL for 0–5 h. **b** 7-AAD staining of primary hepatocytes treated as under (**a**). The percentage (%) of cells in the subG1 cell cycle stage (apoptotic cells) are shown. **c** Morphology of living and apoptotic primary hepatocytes treated with TNFα and FasL as indicated, observed under an inverted phase-contrast microscope, scale bar: 100 µm. Caspase-3/-7 activity in total cell lysates of (**d**) Hepa1-6 or (**e**) AML12 cell lines either untreated, treated with TNFα or FasL alone or pretreated with TNFα for 14 h followed by FasL for 4 h. a.u. arbitrary units. (a, b) Values represent the means of at least three independent experiments ± SD.  (**d**, **e**) Data were obtained from four (**d**) or three (**e**) independent experiments; the horizontal line represents the mean

We previously reported that pretreatment of primary mouse hepatocytes with TNFα for 12 h significantly enhanced FasL-induced caspase-3 activation (Fig. 1a)^28^ and apoptosis (Fig. 1b, c). This was also observed in mouse hepatoma Hepa1-6 cells, immortalized mouse AML12 hepatocytes and primary and SV40-immortalized mouse embryo fibroblasts (MEFs). They all exhibited increased caspase-3 activity (Fig. 1d, e and Supplementary Figure 1b, c) and apoptosis (Supplementary Figure 1a) when they were treated with TNFα 14 h before FasL addition for 4 h. Importantly, TNFα itself did neither induce caspase-3 activity nor apoptosis in any cell type investigated (Fig. 1c–e and Supplementary Figure 1a–c). Thus, TNFα sensitization of FasL-induced apoptosis is a general phenomenon observed in different cell types.

### Fas is upregulated in response to TNFα treatment

**Fig. 2:**
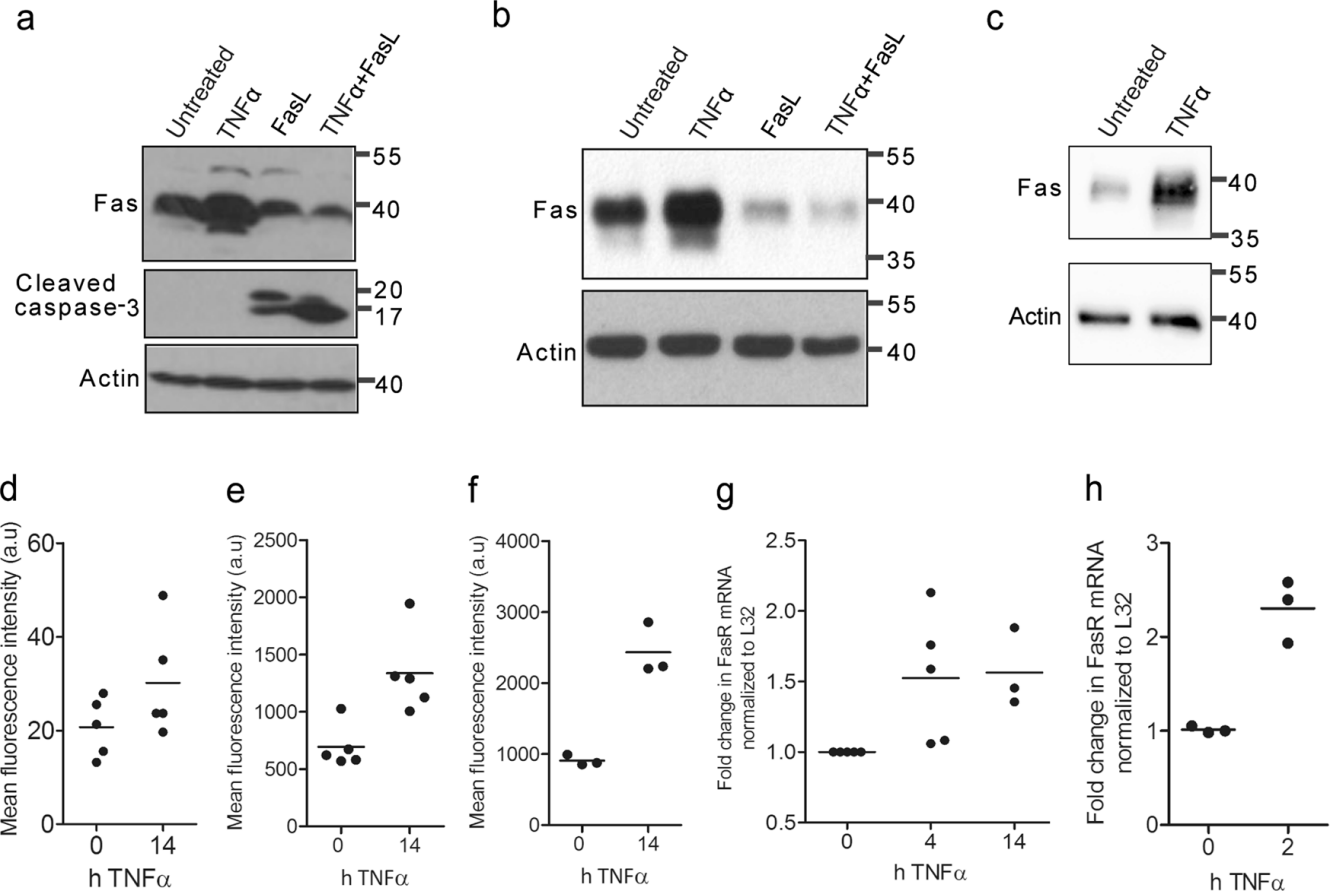
TNFα treatment leads to increased cell surface Fas expression through transcriptional induction. Anti-Fas or anti-cleaved caspase-3 western blot analysis of whole lysates of **a** primary mouse hepatocytes, **b** Hepa1-6 or **c** AML12 cells, either untreated, treated with TNFα or FasL alone or pretreated with TNFα for 14 h before adding FasL for 4 h. Actin serves as loading control. Cell surface expression of Fas measured by FACS analysis using the anti-mFas antibody (conjugated to Alexa Fluor 488) significantly increases after 14 h of TNFα treatment in **d** primary mouse hepatocytes, **e** Hepa1-6 or **f** AML12 cells. RT-qPCR analysis of Fas mRNA (shown as fold change normalized to L32 mRNA) of **g** primary mouse hepatocytes or **h** Hepa1-6 cells shows that Fas gene transcription increases after TNFα treatment for 2 h or 14 h. a.u. arbitrary units. (**d**-**h**)  Data were obtained from five, four or three independent experiments as indicated; the horizontal line represents the mean

The apoptosis sensitization effect of TNFα was dependent on the preincubation of TNFα for >2 h before FasL addition^28^. A simultaneous cellular stimulation with TNFα and FasL did not enhance Fas-induced apoptosis. This indicated that some factor was needed to be induced before the cells became more sensitive to FasL-induced cell death. As TNFα was reported to increase Fas receptor expression in other cell lines^29–33^, we hypothesized that this mechanism may also play a role in hepatocytes. Indeed, we observed increased Fas protein expression after 14 h of TNFα treatment in primary, Hepa1-6 and AML12 hepatocytes (Fig. 2a–c). By contrast, Fas levels diminished after FasL and TNFα/FasL treatments, probably due to endosomal degradation after receptor activation and/or apoptosis induction as seen by increased caspase-3 processing (Fig. 2a, b). Quantitative reverse transcriptase-PCR analysis revealed that increased Fas expression by TNFα was due to increased Fas gene transcription, which was already maximal after 2 h of TNFα treatment (Fig. 2g, h). Moreover, we could show by fluorescence-activated cell sorting (FACS) analysis using an anti-Fas antibody detecting the extracellular domain of Fas that two- to threefold more Fas was expressed on the surface of primary hepatocytes (Fig. 2d), Hepa1-6 (Fig. 2e) and AML12 (Fig. 2f) cells after 14 h of TNFα treatment. Fas mRNA (Supplementary Figure 2a), total protein (Supplementary Figure 1d, e) and surface expression levels (Supplementary Figure 2b) also increased after TNFα treatment in primary and immortalized MEFs.

### Injection of TNFα or LPS trigger Fas upregulation in the liver

**Fig. 3:**
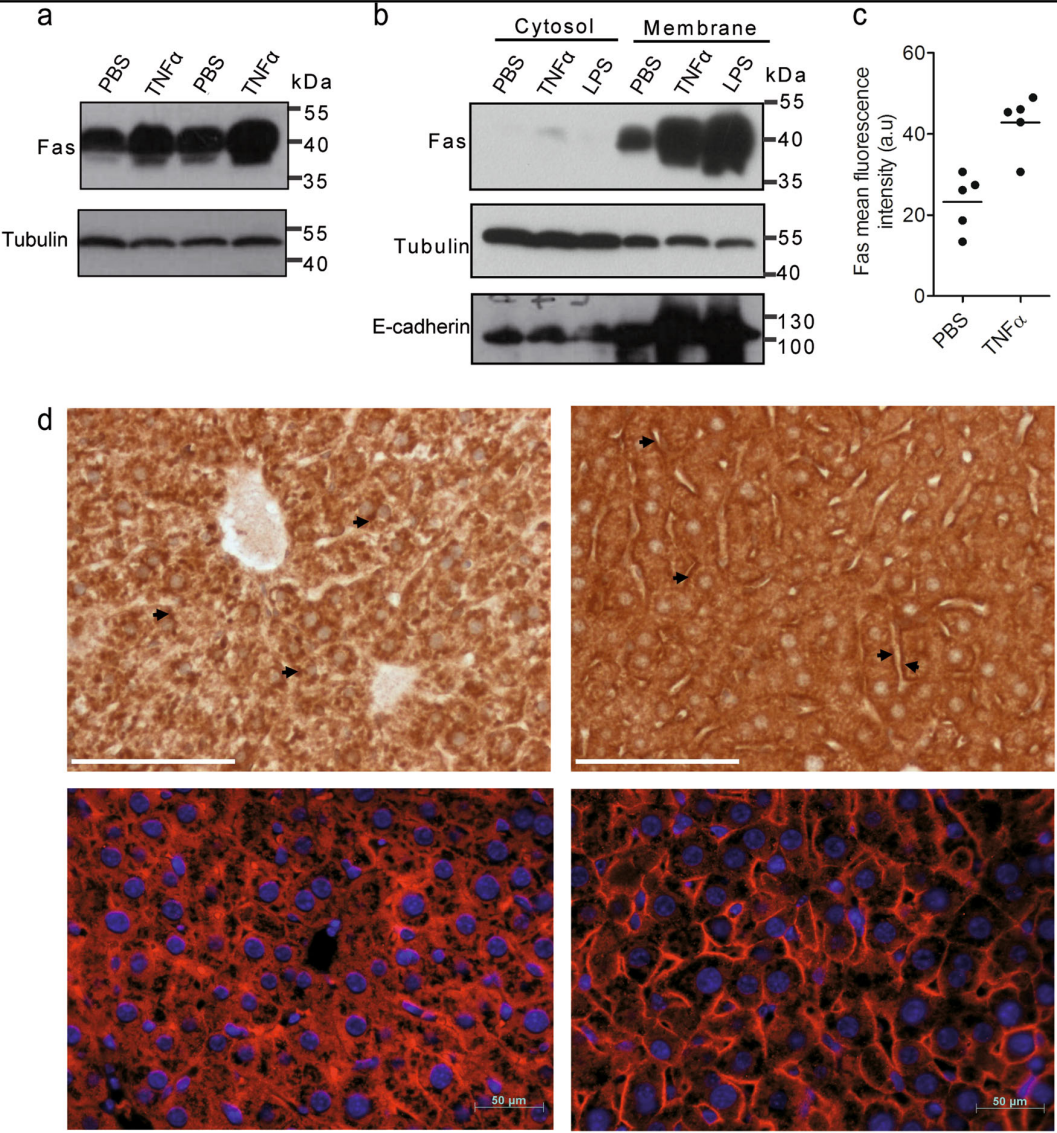
Fas is induced in hepatocytes in the liver after TNFα or LPS injection into mice. **a** Western blot analysis of Fas levels in total liver lysates from two different mice injected with PBS or TNFα for 14 h. **b** Western blot analysis of Fas expression in the cytosol and membrane fractions of whole liver extracts from mice injected with PBS, TNFα or LPS for 14 h. Actin and tubulin serves as loading markers, E-cadherin as marker for plasma membrane proteins. **c** FACS analysis of Fas cell surface expression on hepatocytes freshly isolated from the liver of mice injected with PBS or TNFα for 14 h. Data were obtained from five independent experiments. The horizontal line represents the mean. **d** Anti-mFas DAB (top panels, scale bar: 75 µm) or red immunofluorescence (lower panels, scale bar: 50 µm) staining of liver slices from mice injected with PBS (left panels) or TNFα (right panels) for 14 h. Nuclei are stained with DAPI in the lower panels. Arrows point to the increased surface Fas expression after TNFα treatment

To investigate if TNFα also triggered increased Fas surface expression on hepatocytes in vivo, we injected mice with 200 µg of TNFα/kg of body weight, 2 mg of LPS/kg of body weight or phosphate-buffered saline (PBS) (as control) and sacrificed the animals after 14 h. We noted increased Fas protein expression in total liver lysates from TNFα-treated as compared with PBS-treated animals (Fig. 3a). Most of the Fas protein was found in the membrane fraction (Fig. 3b). Similarly, LPS was capable of inducing membrane-bound Fas in hepatocytes in vivo. We then isolated primary hepatocytes from these mice by collagenase perfusion and subjected them to FACS analysis using an antibody recognizing the extracellular domain of Fas. As shown in Fig. 3c, surface expression of Fas significantly increased on hepatocytes from TNFα-treated as compared with PBS-treated animals. Concomitantly, immunohistochemical analysis on liver sections from TNFα-treated mice revealed an enhanced plasma membrane staining of Fas on hepatocytes (Fig. 3d).

### Knockdown of p65 reduces Fas expression and abrogates apoptosis sensitization by TNFα

**Fig. 4:**
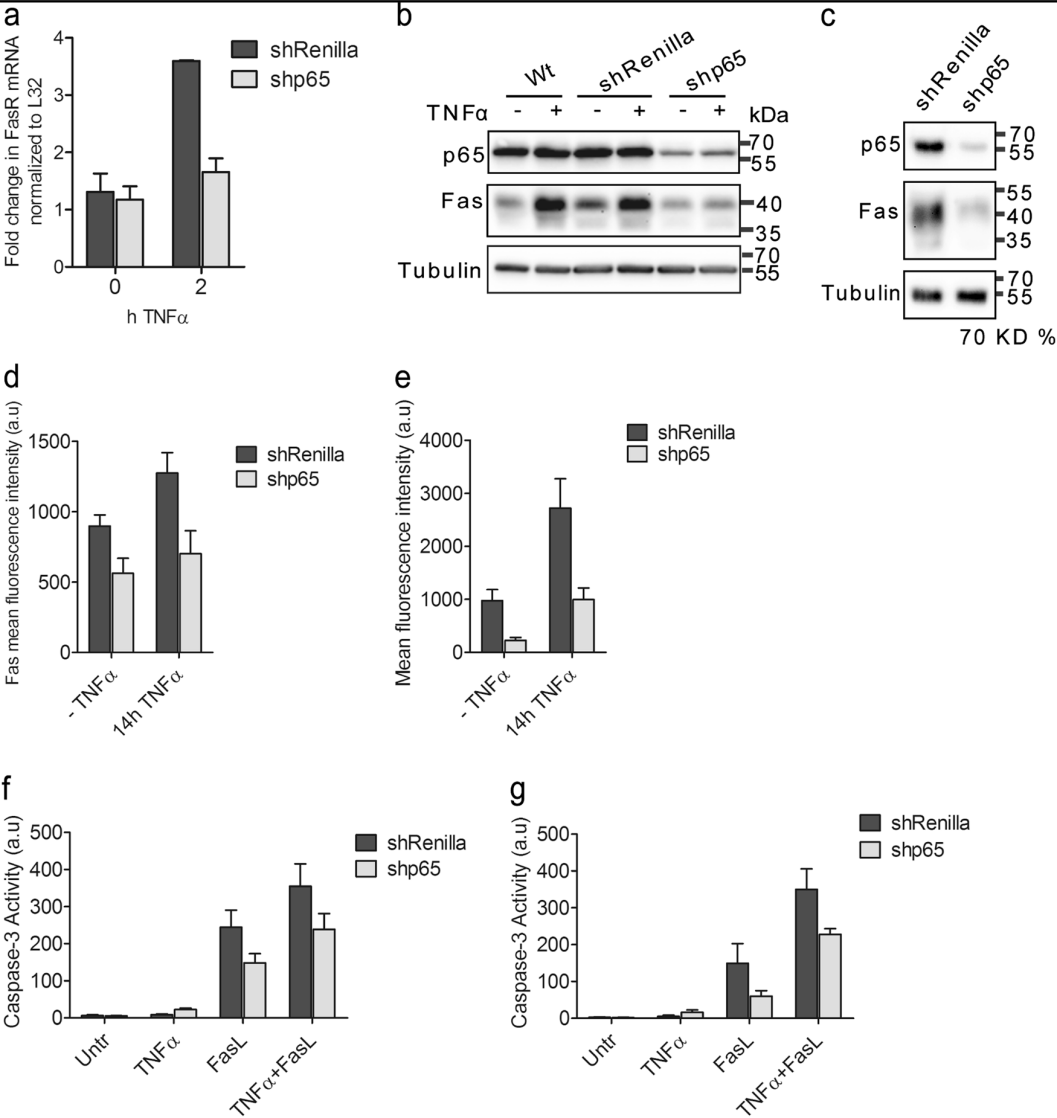
Knockdown of p65 decreases Fas expression and reduces TNFα-mediated sensitization to FasL-induced apoptosis. **a** RT-qPCR analysis of Fas mRNA of Hepa1-6 cells stably expressing Renilla or p65 shRNAs, untreated or treated with TNFα for 2 h. **b** Anti-p65 and anti-Fas western blot analysis of wild-type (Wt), shRenilla or shp65 Hepa1-6 cells, untreated or treated with TNFα for 14 h. Tubulin serves as loading control. **c** The same analysis as in **b** but in AML12 cells. FACS analysis of Fas cell surface expression on shRenilla or shp65 Hepa1-6 (**d**) or AML129 (**e**) cells, untreated or treated with TNFα for 14 h. Caspase-3/-7 activity in total cell lysates of shRenilla or shp65 Hepa1-6 cells (**f**) or AML12 cells (**g**). a.u. arbitrary units. (**a**, **d**-**g**) Values represent the means of at least three independent experiments ± SD

It is well known that TNFα activates the NFκB signaling pathway leading to the transcriptional induction of pro-survival and pro-inflammatory genes. However, previous studies also indicated the induction of apoptotic genes such as Fas by NFκB^31,33^. As expected, the NFκB pathway was quickly activated within 10 min of TNFα treatment in primary mouse hepatocytes (Supplementary Figure 3a) and Hepa1-6 cells (Supplementary Figure 3c) as shown by the phosphorylation and subsequent degradation of the NFκB inhibitor IκBα and the enhanced phosphorylation of the NFκB subunit p65. At 6–8 h posttreatment with TNFα, we also observed a time-dependent increase in total Fas protein expression in both cell lines (Supplementary Figure 3b, c). To elucidate whether Fas was transcriptionally activated by NFκB, we knocked down the expression of the essential p65 subunit of NFκB. For that purpose, we used the microRNA (miR)-adapted small hairpin (sh) RNA (shRNA-miR) system, in which synthetic shRNAs targeting p65, Fas and Renilla luciferase (as control) were embedded in the context of an optimized variant of the existing miR-30 endogenous microRNA (miR-E)^34^. The knockdown efficiency of different p65 shRNAs at single-copy conditions was previously validated^35^, and that of Fas shRNAs is shown in Supplementary Figure 4a. The most potent shRNAs were then chosen to infect Hepa1-6 and AML12 cells. The knockdown efficiency of both p65 NFκB and Fas shRNAs was up to 70% (Fig. 4b, c and Supplementary Figure 4b, d). Importantly, downregulation of p65 was accompanied by reduced transcription of canonical NFκB-target genes IκBα and Bcl-x_L_ (Supplementary Figure 4d). Also, FasL- and TNFα/FasL-induced caspase-3 activity was largely diminished in the presence of shFas (Supplementary Figure 4c) indicating that the shRNAs effectively reduced the biological function of their respective targets.

Although TNFα still induced a fourfold increase of Fas mRNA in shRenilla control Hepa1-6 cells as compared with untreated cells, this effect was entirely ablated when p65 expression was downregulated by p65 shRNA (Fig. 4a). Similarly, Fas protein levels were not augmented by TNFα upon p65 reduction in both Hepa1-6 (Fig. 4b) and AML12 cells (Fig. 4c). This was also the case for Fas surface expression. Introduction of shp65 into Hepa1-6 and AML12 cells completely prevented the increase of Fas protein on the cell surface after 14 h of TNFα treatment (Fig. 4d, e). In both cell lines, the failure to augment surface Fas expression diminished TNFα sensitization of FasL-induced caspase-3 activation to the level seen with FasL alone (Fig. 4f, g) indicating that the apoptosis sensitizing effect of TNFα required the NFκB-mediated transcriptional induction and surface expression of Fas. Interestingly, even in the absence of TNFα, surface Fas expression and FasL-induced caspase-3 activity was diminished after p65 knockdown (Fig. 4b–g) suggesting that Fas expression was controlled by NFκB even under steady-state conditions.

To further substantiate the role of p65 NFκB in the Fas upregulation and apoptosis sensitization by TNFα, we used MEF cell lines either deficient of p65 (p65 KO) or expressing a dominant-negative (DN) form of the NFκB inhibitor IκBα, which cannot be phosphorylated and subsequently degraded. 3T9- or SV40-transformed wild-type (WT) MEFs showed a marked increase in mRNA (Supplementary Figure 2a), protein (Supplementary Figure 2d) and surface expression of Fas (Supplementary Figure 2b) and were sensitized to FasL-induced caspase-3 activation after TNFα (pre-) treatment for 14 h (Supplementary Figure 2c). All these effects were drastically diminished or even ablated when p65 was missing (p65 KO) or inhibited by IκBαDN. Again, total Fas protein and surface expression were already reduced in IκBαDN cells or p65 KO in the absence of TNFα.

### HepG2 cells do not upregulate Fas in response to TNFα and are therefore not sensitized to FasL-induced apoptosis

**Fig. 5:**
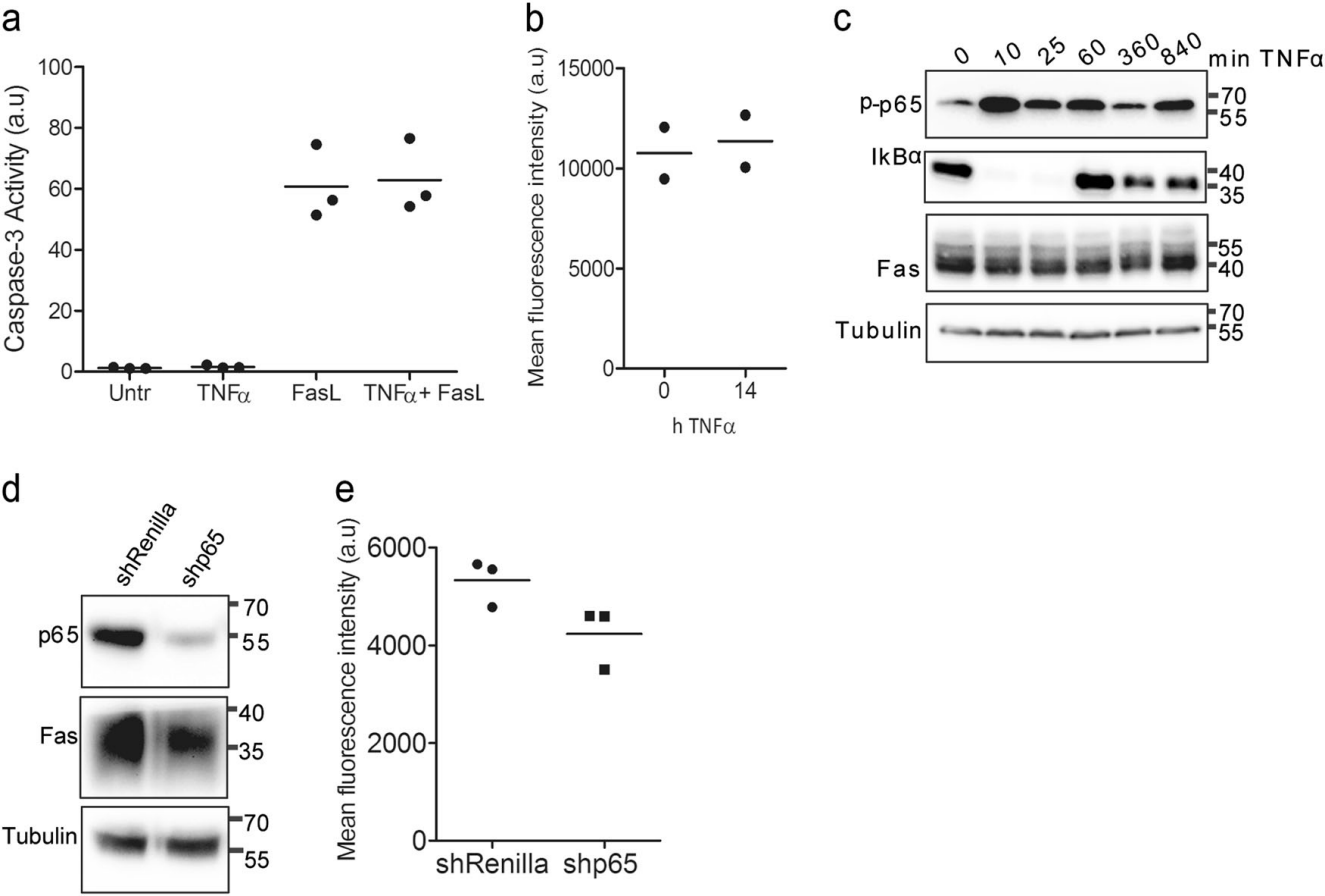
TNFα treatment does neither upregulate Fas nor enhance FasL-induced apoptosis in HepG2 cells. **a** Caspase-3/-7 activity in total lysates of HepG2 cells, either untreated, treated with TNFα or FasL alone or pretreated with TNFα for 14 h before adding FasL for 4 h. **b** FACS analysis of surface Fas expression in HepG2 cells treated with TNFα for 14 h. a.u. arbitrary units. **c** Anti-phospho-p65, anti-IκBα and anti-Fas western blot analysis of total extracts of HepG2 treated with TNFα for up to 840 min. **d** Anti-p65 and anti-Fas western blot analysis of total extracts of shRenilla or shp65 HepG2 cells. Tubulin serves as loading controls. **e** FACS analysis of Fas surface expression using anti-hFas conjugated to APC on shRenilla or shp65 HepG2 cells. (**a**, **b**, **e**) Data were obtained from three or two independent experiments as indicated. The horizontal line represents the mean

To corroborate the connection between TNFα-induced Fas upregulation and sensitization to FasL-induced apoptosis, we performed our studies in the human hepatoblastoma cell line HepG2. Surprisingly, TNFα treatment did not sensitize HepG2 cells to FasL-induced apoptosis (Fig. 5a). Total Fas protein and surface expression were already high in HepG2 cells and TNFα was incapable of further increasing its level (Fig. 5b, c), despite normal activation of NFκB (Fig. 5c). Moreover, knockdown of p65 by shRNA expression only slightly diminished the amount of cellular Fas (Fig. 5d, e) indicating that in this cancer cell line Fas expression was not under the control of NFκB and hence TNFα was unable to sensitize the cells to FasL-induced apoptosis. These results indicate that TNFα sensitization to FasL-induced apoptosis is tightly linked to Fas upregulation.

### Knockdown of p65 in vivo reduces TNFα-mediated Fas upregulation in the mouse liver

**Fig. 6:**
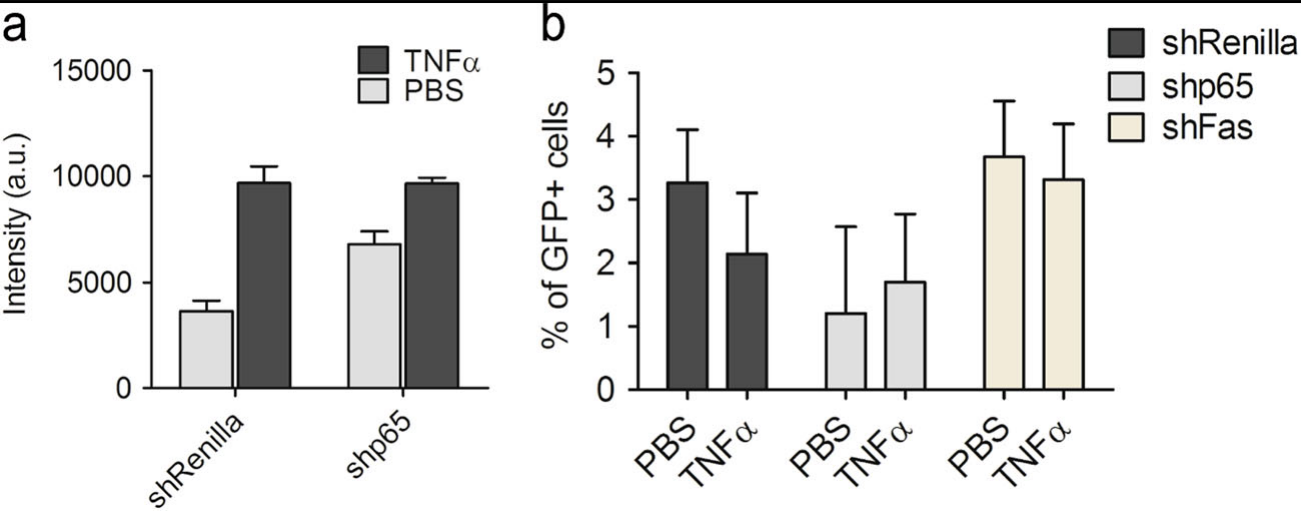
Knockdown of p65 in hepatocytes in vivo decreases TNFα-mediated Fas upregulation. FACS analysis of primary hepatocytes isolated from mice tail vein injected with plasmids carrying the p65, Fas or Renilla shRNAs for 5 days and treated with PBS or TNFα for 14 h, stained with anti-Fas-APC or isotype-APC. **a** GFP-positive cells, which incorporated the shRenilla (gray bars) or p65 shRNAs (black bars) were separately gated to calculate the mean fluorescence of Fas surface expression; a.u. arbitrary units. **b** Percentage of isolated, GFP-positive hepatocytes that incorporated the respective injected shRNAs (shRenilla, black; shp65, gray; shFas, dark gray). Values represent the means of at least three mice ± SD.

To test the role of NFκB for TNFα-induced Fas upregulation in vivo, a recombinant Sleeping Beauty (SB) transposon vector harboring p65, Renilla (negative control) or Fas shRNAs and a vector containing a transposase were combined and co-injected into mice by hydrodynamic tail vein injection. This procedure allows the rapid delivery of the shRNAs through the lateral tail vein into the liver and their selective uptake by hepatocytes^36^. The transient expression of the transposase facilitates the integration of the transposons into the hepatocytes DNA allowing stable transgene expression. Due to coupling green fluorescent protein (GFP) to the shRNA, hepatocytes expressing the respective shRNA can be easily monitored by green fluorescence microscopy. Five days after hydrodynamic tail vein injection with the different shRNAs, the mice were injected intraperitoneally with PBS or TNFα and sacrificed after 14 h. As shown in Supplementary Figure 5a, about 5–10% of hepatocytes had taken up the shRNAs (GFP-positive hepatocytes). Anti-p65 immunofluorescence analysis of liver slices of mice injected with p65 shRNA revealed that the GFP-positive cells exhibited an efficient downregulation of p65 NFκB expression (Supplementary Figure 5a, comparing upper and lower panels). As previously shown (Fig. 3d), TNFα treatment for 14 h increased Fas surface levels on hepatocytes in vivo (Supplementary Figure 5b). However, by this analysis it was difficult to determine if Fas surface expression was diminished in shp65/GFP-positive hepatocytes due to the proximity of high surface Fas expressing, GFP-negative hepatocytes (Supplementary Figure 5b). We therefore isolated hepatocytes from Renilla, p65 or Fas shRNA tail-injected animals after TNFα- or PBS treatment and subjected them to FACS analysis using GFP (green) and anti-Fas-APC (red) fluorescence analysis. Consistent with our previous findings, the GFP-positive hepatocytes from mice injected with shRenilla control plasmids showed a significant increase in Fas surface levels after TNFα treatment as compared with those isolated from PBS-treated animals (Fig. 6a, gray bars). This was not the case for GFP-positive hepatocytes carrying the shp65 plasmid. Here TNFα treatment did not achieve a further upregulation of Fas surface expression as compared with PBS-treated animals (Fig. 6a, black bars). Of note, we found less GFP-positive cells in the liver of shp65-injected as compared with shRenilla-injected or shFas-injected mice (Fig. 6b), most likely because the lack of p65 may be detrimental to the survival of liver cells and therefore lead to their cell death. In summary, we could prove that NFκB contributes to Fas surface upregulation in response to TNFα not only in vitro but also in murine livers in vivo.

## Discussion

We previously reported that cell pretreatment with TNFα for a minimum of 12 h sensitized murine primary hepatocytes to FasL-induced apoptosis via JNK activation and phosphorylation/activation of Bim^28^. Here we uncover an additional mechanism for this sensitization. We find that primary murine hepatocytes, as well as established hepatocyte and embryo fibroblast cell lines upregulate Fas in response to TNFα in an NFκB-dependent manner. The p65 subunit of NFκB was not only required for TNFα-induced Fas transcription, but also for Fas protein and Fas cell surface expression. Fas transcription even seemed to depend on p65 in unstimulated cells (in the absence of TNFα) suggesting that p65 might be the major transcription factor driving Fas surface expression in hepatocytes and other cells. Importantly, this mechanism also occurred in the liver in vivo, as tail vein injection of mice with shp65 diminished TNFα-induced upregulation of Fas surface expression on isolated primary hepatocytes. Although we could clearly show that p65-mediated Fas upregulation sensitized hepatocytes and fibroblasts for FasL-induced apoptosis in vitro, this was difficult to confirm in vivo. Mice injected with FasL or agonistic anti-Fas antibodies die within 4 h due to liver failure. A pre-injection of mice with TNFα or LPS is expected to kill these animals even faster, a condition that cannot be approved under the animal experiment regulations in Germany.

Our findings partly explain studies by other authors in the past. For example, Costelli and colleagues reported in 2003 that mice deficient in TNFR1 and TNFR2 were resistant to a single dose of anti-Fas that was lethal for TNFR-expressing animals^26^. They claimed that treatment with anti-Fas somehow engaged the TNFα/TNFR system, which synergized with Fas-mediated signals to cause fulminant liver injury. In agreement with these results, Matsuki and colleagues provided evidence that injection of anti-Fas antibodies triggered the production of TNFα by Kupffer cells in the liver^37^. Moreover, full protection from anti-Fas-induced mortality could only be achieved by pretreatment with neutralizing antibodies against Fas and TNFR1. Furthermore, in an experimental mouse model of alcohol-induced liver injury, Minagawa and colleagues showed that chronic alcohol feeding led to an increase in NKT cells in the liver that mediated hepatocyte cell death via the Fas pathway. Also here TNFα seemed to play a crucial role as TNFR1-deficient animals were protected from Fas-induced liver injury^25^. Besides the direct alcohol injury on the liver, excessive alcohol uptake impairs the gut barrier function leading to elevated endotoxin levels in the liver. In this scenario, LPS stimulates the release of TNFα by local macrophages (Kupffer cells), which then acts on hepatocytes to upregulate Fas and amplify FasL-induced cell death. In this context, it is reasonable to postulate that under certain conditions of liver inflammation, TNFα-mediated Fas upregulation may be a critical factor for the amplification of hepatocyte apoptosis in vivo. In addition, our immunohistochemistry (IHC)/immunofluorescence analysis suggest that TNFα may not only trigger the upregulation of the Fas protein but also its transport from intracellular compartments to the cell surface. It has been demonstrated that in unstimulated cells, Fas is mainly stored in the cytoplasm particularly in the Golgi^38,39^.

TNFα potently activates NFκB via phosphorylation-dependent, proteasomal degradation of its inhibitor IκB. Active NFκB then induces the expression of a variety of anti-apoptotic and pro-inflammatory genes^17,40^. Paradoxically, NFκB was also reported to influence the expression of pro-apoptotic genes such as ligands and receptors of the TNF superfamily^41^ but until now the effect of NFκB activation on the apoptosis sensitivity of primary hepatocytes has not yet been investigated. In contrast to NFκB activation (based on IκB phosphorylation/degradation and p65 phosphorylation), which occurs within 10–30 min of TNF treatment, Fas upregulation and concomitant sensitization to FasL-induced apoptosis takes 6–12 h to proceed (Supplementary Figure 3). Two distinct waves of NFκB recruitment to target promoters have been reported: a fast recruitment to constitutively and immediately accessible promoters and a late recruitment to promoters requiring stimulus-dependent modifications in chromatin structure to make NFκB sites accessible^42^. NFκB-regulated pro-apoptotic genes like Fas may be under the control of late recruitment promoters explaining the delayed transcriptional Fas upregulation observed in response to TNFα in our cellular systems. An example that integrates the protective role of NFκB activity and the enhancement of cell sensitivity to apoptosis may be found in pre-malignant senescent hepatocytes. Senescent cells constitutively activate NFκB, which leads to the so-called senescence-associated secretory phenotype^43^. This phenotype includes the release of inflammatory cytokines, which serves to recruit T lymphocytes and NK cells for the clearance of the senescent cells. It may be possible that this constitutive NFκB activation contributes to overexpression of Fas that helps to eliminate the senescent cells by FasL-expressing T lymphocytes. As FasL is mainly produced by activated immune cells, hepatocytes and other cells expressing high levels of Fas, either in a constitutive or TNFα-inducible manner, are not immediately killed but only when recognized and/or attacked by immune cells.

In our in vivo knockdown experiments, we found a smaller increase of Fas surface expression in GFP+ cells from p65 shRNA injected mice as compared with shRenilla injected mice after TNFα treatment. This tendency is comparable to our in vitro results. However, the yield of isolating GFP+ primary hepatocytes from the shp65 injected mice was significantly lower compared with the shRenilla and shFas-injected animals. This indicates that knocking down p65 could have been cytotoxic to hepatocytes in vivo. This would be consistent with the previous finding that NFκB is an important survival factor in liver cells as mice lacking the p65 subunit of NFκB exhibit embryonic lethality at days 15–16 of gestation, accompanied by massive destruction of liver via apoptosis^44^. In this respect, it was advantageous for our studies that p65 shRNA was not expressed in all hepatocytes after tail vein injection and did not completely knockout the expression of p65 in these cells.

TNFα is known to induce both NFκB and JNK signaling. Although JNK has been mostly associated with apoptotic outcome, NFκB activation predominantly ensures cell survival. We previously published that JNK activation and subsequent JNK-mediated Bim phosphorylation at three sites enhances the pro-apoptotic activity of Bim toward Bax/Bak activation and contributes to TNFα sensitization of FasL-induced apoptosis^28^. However, blocking this pathway by JNK inhibition or genetic ablation of Bim did not fully inhibit the TNF sensitization. We now show that also NFκB-mediated FasL upregulation contributes to this process. Thus, NFκB does not only control hepatocytes survival but also their sensitivity of FasL-induced cell death. Our finding will further enlarge the molecular understanding of various types of liver diseases and will eventually contribute to the development of novel therapies.

## Materials and methods

### Cell lines

WT and p65-/- embryonic fibroblasts (MEF) immortalized with SV40 T antigen were kindly provided by Andreas Strasser (WEHI, Melbourne, Australia), HEK293T and HepG2 (ATCC, Manassas, VA, USA), mouse hepatoma Hepa1-6 and 3T3 MEFs were cultured in Dulbecco’s modified Eagle’s medium (DMEM) high glucose medium, supplemented with 10% fetal calf serum and 1% penicillin/streptomycin. AML12 mouse hepatocytes were cultured in William's medium supplemented with 100 nM dexamethasone, 2 mM l-glutamine, insulin 1 µM, 10% fetal calf serum and 1% penicillin/streptomycin.

### Cell treatments

Primary mouse hepatocytes were incubated with medium containing TNFα (20 ng/ml, Peprotech, London, UK) or not for 14 h. The cells were either harvested for Fas expression analysis, or Fc-FasL (20 ng/ml, Adipogen, Epalinges, Switzerland) was added to measure cell death. The other cell lines were plated on six-well plates at a density of 2.5 × 10^5^ cells/well 1 day before treatment. On the next day, they were treated in the same way as primary mouse hepatocytes. HepG2 cells were treated with human instead of mouse TNFα.

### Animal studies

All C57BL/6 mice were kept under specific pathogen-free conditions and a controlled humidity and lighting schedule with a 12-h light/dark period and free access to food (regular mouse chow) and water. Mouse experiments were performed in Freiburg, Tübingen and New York, complied with animal experimentation regulations of Germany and the US and were approved by the Ethics Review Committee of the regional Council in Tübingen/Freiburg and the Memorial Sloan Kettering Cancer Center (MSKCC) Animal Care and Use Committee in New York City.

### Isolation and culture of primary mouse hepatocytes

Primary mouse hepatocytes were isolated from 8 to 14 weeks old male C57BL/6 mice (Jackson Laboratories, Bar Harbor, ME, USA) by collagenase perfusion as reported^45^. Briefly, the mice were anesthetized by intraperitoneal (i.p) injection with 5 mg 10% ketamine hydrochloride (Ketanest, Pfizer, NY, USA) and 1 mg 2% xylazine hydrochloride (Rompun, Bayer, Leverkusen, Germany) per 100 g of body weight. The abdominal cavity was opened and the portal vein cannulated with a 24G catheter connected to a peristaltic pump through a silicon tube. The liver was perfused with Hanks solution I (Hanks, 2 mM EGTA, 0.1% glucose) for 2 min at a flow rate of 8 ml/min. Afterward, Hanks solution II was perfused (Hanks, 5 mM CaCl_2_, 0.3 mg/ml collagenase CLS II) for 5–7 min and the liver was removed from the mouse. The liver capsule was taken with forceps and carefully shaken until the hepatocytes were released. The cells were filtered through a 70 μM cell strainer and washed in William´s medium supplemented with 100 nM dexamethasone, 2 mM l-glutamine, 1 µM insulin, 10% fetal calf serum and 1% penicillin/streptomycin (attachment medium) twice (50 × *g* for 2 min centrifugation). The hepatocyte suspension was either used right after isolation (e.g., Fas receptor analysis) or plated (1 × 10^6^ cells/well) on rat tail collagen I-coated six-well plates in attachment medium. After allowing the cells to attach for 4 h, they were washed and further incubated in William´s medium supplemented with 2 mM l-glutamine, 10% fetal calf serum and 1% penicillin/streptomycin. Primary hepatocytes were used for experiments within 2–3 days after isolation.

### RNA extraction and RT-qPCR

Total RNA was isolated from non-treated and TNFα-treated cells using the TRIZOL reagent (Thermo Fisher Scientific, Darmstadt, Germany). Reverse transcription was performed using 2 µg of total RNA (Invitrogen Superscript First Strand Synthesis Kit, Invitrogen, Karlsruhe, Germany) plus random hexamers. Quantitative PCR (qPCR) was performed in 96-well plates using 12.5 µl Mesa Blue qPCR Mix (including Hotstar meteor Taq polymerase), 0.5 µl of each forward and reverse primer for the candidate gene (10 µM stock), 9.5 µl of distilled H_2_O and 2 µl of the reverse-transcribed complementary DNA (cDNA) per well. The housekeeping gene L32 was used for normalization. For each cDNA sample, triplicates were measured with the two primer pairs (gene of interest and housekeeping gene). The plate was sealed with adhesive foil, spun down at 1000 rpm for 1 min and then subjected to the PCR reaction in a Biorad MyiQ Real-time PCR machine (Bio-Rad, Munich, Germany).

### Preparation of whole-cell lysates

For the preparation of whole-cell lysates, the cells were harvested, washed once with ice‐cold PBS and then lysed in extraction buffer [20 mM Tris–HCl, pH 7.5, 150 mM NaCl, 1% Triton X‐100, 5 mM EDTA, 1 × protease inhibitor cocktail complete (Roche, Penzberg, Germany), MG132 (20 μM, Alexis Biochemicals, Laussane, Switzerland), phosphatase inhibitor cocktail 1 (1:50, Sigma‐Aldrich)] on ice for 15 min. Lysates were centrifuged at 13,000 rpm for 5 min at 4 °C. For the subcellular fractionation of liver samples, a piece of liver was cut and washed several times in PBS to eliminate the red blood cells. The tissue was first homogenized in RIPA buffer + 0.2% NP-40 for 10 min using a Dounce homogenizer on ice. The extract was centrifuged at 20,000 *g* for 20 min and the supernatant was kept as the cytosolic fraction. The pellet was further lysed in RIPA buffer + 1% sodium dodecyl sulfate (SDS) for 30 min on ice and then centrifuged at 20,000 *g* for 20 min. The supernatant was considered as solubilized membrane fraction. The protein concentration was determined using the bisinchoninic acid protein assay (BCA, Pierce Thermo Scientific, Darmstadt, Germany).

### Immunoblot analysis and antibodies

For western blot analysis, the cellular extracts were solubilized in 3× Laemmli buffer consisting of 1% SDS and boiled for 5 min at 95 °C. Equal protein amounts (30–80 μg) were separated on SDS–polyacrylamide gel electrophoresis (12.5–15% gels) and transferred to nitrocellulose membranes. Antibody detection was accomplished using the enhanced chemiluminescence method (Thermo Fisher Scientific, Darmstadt, Germany) and developed either with the Fusion SL Imager (Vilber Lourmat, Eberhardzell, Germany) or the Curix60 processor (Agfa healthcare, Bonn, Germany). The following antibodies were used in 3% milk/TBS–Tween (0.1%): anti-mFas (1:1000, Santa Cruz Biotechnology, Dallas, TX, USA), anti-actin (1:40,000, MP Biomedicals, Eschwege, Germany), anti-tubulin (Bio-Rad, Munich, Germany), anti-E-cadherin (BD Transduction laboratories, Heidelberg, Germany), anti-hFas, anti-p65, anti-phospho-p65, anti-IκBα, anti-phospho-IκBα, anti-cleaved caspase-3 and anti-Bcl-x_L_ (Cell Signaling, Leiden, The Netherlands).

### Cell death assays

To determine the enzymatic activity of effector caspase-3 and caspase-7, 40 µg of a whole-cell lysate was incubated with the fluorogenic substrate DEVD‐AMC (60 μM, Enzo Life Science, Lausen, Switzerland) in caspase-3 activity buffer (100 mM Hepes, pH 7.5, 10 mM dithiothreitol). Right afterward, fluorescence (460 nm) was measured at 1-min intervals during 30 min (37 °C, Tecan Infinite M200 microplate reader, Crailsheim, Germany). The relative caspase activity (arbitrary units) was calculated as the slope of the linear regression between the absorbance and the time. Cell death in Hepa1-6 and MEFs cells was further determined by FACS analysis (FACSDiva, BD Biosciences, Heidelberg, Germany) using the Annexin-V/7-AAD detection system. Cells were harvested and washed in Annexin binding buffer (10 mM Hepes, 140 mM NaCl, 2.5 mM CaCl_2_, pH 7.4) and stained for 15 min in the same buffer with Annexin-V-FITC (1 µg/ml, produced in-house). The DNA dye 7-aminoactinomycin D (7-AAD) (1 µg/ml, Thermo Fisher Scientific, Darmstadt, Germany) was added right before measurement. In the case of primary mouse hepatocytes, viability was assessed by DNA content analysis, considering the subG1 peak as the dying population. For that purpose, the hepatocytes were fixed with 70% ethanol, washed with PBS and then stained with a solution of propidium iodide (PI) (20 µg/ml, Sigma-Aldrich, Taufkirchen, Germany) containing RNAase (200 µg/ml, Qiagen, Hilden, Germany).

### shRNA design and cloning

De novo shRNAs predictions for p65 and Fas targeting common regions of all known transcript variants were obtained using the SplashRNA algorithm. The existing miR-30 p65-1 shRNA was converted to miR-E by PCR amplification using the miRE-Xho-short-fw (5′-AGAAGGCTCGAGAAGGTATATTGC-3′) and miR-E EcoPlasmid-rev (5′-GCTCGAATTCTAGCCCCTTGAAGTCCGAGG-3′) primers. For de novo generation of miR-E shRNAs, 97-mer oligonucleotides (IDT Ultramers, Coralville, IA, USA) coding for p65 and Fas shRNAs were PCR amplified using the primers miRE-Xho-fw (5′-TGAACTCGAGAAGGTATATTGCTGTTGACAGTGAGCG-3′) and miRE-EcoOligo-rev (5′-TCTCGAATTCTAGCCCCTTGAAGTCCGAGGCAGTAGGC-3′). The amplification products were cloned into the constitutive retroviral MLP-E vectors (MSCV-LTR-mir-E-PGK-Puro-IRES-GFP). After the ligation, Top10 bacteria were incubated for 15 min on ice and then transformed with the ligation products by heat-shock for 45 s at 42 °C. Afterward, the bacteria were incubated on ice for 10 min and then plated on agar plates containing 100 μg/ml ampicillin. After incubating the plates overnight at 37 °C, three colonies from each ligation were picked up and incubated in 2 ml lysogeny broth medium  as described before overnight. The plasmids were purified the next morning using the QIAprep miniprep DNA Purification Kit (Qiagen, Hilden, Germany). Plasmids containing correct sequences were used to build the retrovirus in order to evaluate the knockdown efficiency of each predicted shRNA in NIH/3T3 MEFs. For hydrodynamic tail vein injections experiments, the shRNAs showing the highest knockdown efficiency were further subcloned into the pT3-EF1a-GFP-miR-E transposon vector using MAX Efficiency Stbl3 competent cells. The DNA was purified with the Qiagen Plasmid *Plus* Midi Kit or the ZymoPure Maxiprep Kit (Zymo Research, Irvine, CA, USA).

### Retrovirus production, infections and transfections

The retroviruses for evaluating the knockdown efficiency of each single shRNA were produced using the retrovirus packaging cell line Plat-E (Cell Biolabs, San Diego, CA, USA). The cells were transfected with 20 μg of the MLP-E plasmids using the calcium–phosphate precipitation method. After 48 h, the virus containing medium was filtered (0.45 μm filter) and supplemented with 4 μg/ml polybrene (Sigma-Aldrich, Taufkirchen, Germany). 3T3 cells were infected under single-copy conditions as previously described^46^. Briefly, different dilutions of viral supernatants were used and the transduction efficiency was assessed by measuring the percentage of GFP-positive cells at 72 h post-infection (Flow cytometry, Guava Technologies, Chicago, IL, USA). The cells showing <20% of GFP positivity were selected in 4 μg/ml of puromycin (Sigma-Aldrich, Taufkirchen, Germany) for 3 days.

To generate retrovirus for infecting Hepa1-6, AML12 and HepG2 cells, 293T cells were transfected either with 3 μg of retroviral MLP-E vectors targeting p65, Fas or control Renilla Luciferase, 3 μg Hit60 and 3 μg pVSV‐G (ClonTech, Mountain View, CA, USA) using polyethylenimine (PEI) transfection^47^. The following day, 5 mM butyrate (Sigma‐Aldrich, Taufkirchen, Germany) was added for 8 h to enhance expression. The supernatants containing the virus were filtered the next day (0.45 μm) and frozen in 5 μg/ml polybrene. For infection, the cells of interest were plated the day before at a 30% confluence on 12-well plates. The next morning, the cells were infected with 400 μl of virus supernatant, spun down for 10 min at 400 *g* and incubated for 6–8 h at 37 °C. This infection protocol was performed three times followed by antibiotic selection using 4 μg/ml puromycin for 4 days.

To express a DN from of IκB (S32A/S36A), SV40-transformed MEFs were seeded in six-well plates (200,000 cells/well) and on the next day transiently transfected with a mixture of pcDNA3-IκB(S32A/S36A) or the pcDNA3 control vector (1.2 µg) plus 4.5 µl of Attractene (Qiagen, Hilden, Germany) in 94.3 µl OptiMEM per well. After 24 h, the cells were used for further analysis.

### Flow cytometry analysis of Fas cell surface expression

The cells were harvested and washed once in PBS/0.5% bovine serum albumin (BSA) buffer following an incubation in the same buffer of 1 h with anti-mFas or an IgG1 isotype control antibody conjugated to Alexa Fluor 488 (GFP-negative cells) or PE or APC (GFP-positive cells) (all at 4 µg/ml, Thermo Fisher, Darmstadt, Germany). The DNA dye 4,6-diamidino-2-phenylindole (DAPI; 50 µg/ml, Sigma-Aldrich, Taufkirchen, Germany) was added right before measurement to exclude dead cells. Flow cytometry analysis was performed using the BD FACS LSRII and FACSDiva.

### Immunohistochemistry and immunofluorescence

Fourteen hours after TNFα or LPS injection, the mice were euthanized with a CO_2_ overdose, liver samples were immediately resected and fixed overnight in 4% paraformaldehyde (Sigma-Aldrich, Taufkirchen, Germany) in PBS. The next steps were performed as previously described^48^. Briefly, the sections were blocked in 5% BSA/PBS for 1 h and then incubated overnight in the same buffer containing one or a combination of the following primary antibodies: anti-mFas (1 µg/ml, Santa Cruz Biotechnology, Dallas, TX, USA), anti-p65 (1 µg/ml, Santa Cruz), anti-GFP (0.5 µg/ml, Abcam, Cambridge, MA, USA) or rabbit immunoglobulin (1 µg/ml, Dianova, Hamburg, Germany) as staining control. For immunohistochemistry, a biotinylated goat anti-rabbit antibody diluted 1:200 in antibody dilution buffer was then added to the sections and incubated in the dark at room temperature for 2 h. The Vectastain ABC kit (Vector Labs, Burlingame, CA, USA) was used for immunoperoxidase amplification and the ImmPACT DAB (diaminobenzidine) substrate to develop the signal. For immunofluorescence, anti-rabbit-Alexa 594 secondary antibody for the detection of mFas and p65 and anti-chicken-Alexa 488 for the detection of GFP-positive cells were used (1 µg/ml, Thermo Fisher Scientific, Darmstadt, Germany). After washing the secondary antibodies, the sections were incubated with Hoechst (1:1000, Sigma-Aldrich, Taufkirchen, Germany) to detect the nuclei and then mounted with ProLong GOLD mounting medium. The tissue was imaged with a fluorescent Microscope (Axiovert 40C, Zeiss, Jena, Germany).

### In vivo experiments and hydrodynamic tail vein injections

Eight to 14 weeks old C57BL/6 mice were injected i.p. with 200 µg of TNFα/kg of body weight or 2 mg of LPS/kg of body weight and sacrificed after 14 h to isolate primary hepatocytes by liver perfusion or to prepare liver tissue sections for IHC. Control animals were i.p injected with PBS. For Fas cell surface, expression analysis isolated suspension hepatocytes were directly measured without plating them on collagen.

For hydrodynamic tail vein injections, a solution/plasmid mix was prepared for each injection containing 20 µg of DNA of pT3-EF1a-GFP-miR-E transposon vector (containing shp65, shFas or shRenilla) together with CMV-SB13 Transposase (1:5 ratio) in sterile 0.9% NaCl^49^. Eight to 14 weeks old female C57BL/6 mice were injected with the 0.9% NaCl solution/plasmid mix into the lateral tail vein with a total volume corresponding to 10% of body weight in 5–7 s. The injections of each shRNA were performed on three different days (10 mice for each shRNA). After 5 days, the mice were i.p injected with PBS or TNFα as mentioned above. After 14 h, the mice were euthanized with a CO_2_ overdose and the livers resected for immunofluorescence or anesthetized for the isolation of hepatocytes by collagenase perfusion (each group isolated on three different days).

## Electronic supplementary material

Supplemental Text

Figure S1

Figure S2

Figure S3

Figure S4

Figure S5

Figure S6
