# Supplementary material for: TNFα sensitizes hepatocytes to FasL-induced apoptosis by NFκB-mediated Fas upregulation
Source: Cell Death Dis. 2018 Sep 5;9(9):909. doi: 10.1038/s41419-018-0935-9 (PMC6125596; doi:10.1038/s41419-018-0935-9)
Supplement: Supplementary file 1 — Supplemental Text [file 41419_2018_935_MOESM1_ESM.pdf]

Supplementary Information

Supplementary Figure 1: TNFα triggers increased Fas protein expression in SV40 immortalized and primary MEFs and sensitizes the cells to FasL-induced apoptosis. (a) Annexin-V-FITC/7-AAD FACS analysis of SV40 immortalized mouse embryonic fibroblasts (MEFs), either untreated, treated with TNFα or FasL alone or pretreated with TNFα 14 h before adding FasL for 4 h. Annexin-V-FITC/7-AAD negative surviving cells are depicted. Less cells survive in response to TNFα/FasL than FasL alone. Caspase-3/-7 activity of total lysates of primary **(b)** or SV40 immortalized MEFs **(c)**, treated as described under (a), showing enhanced protease activity in TNFα + FasL as compared to FasL-treated cells. Values shown are from three independent experiments; the horizontal line represents the mean. Anti-Fas western blot analysis of total extracts of primary **(d)** or SV40 MEFs **(e)**, treated as described under (a), showing increased Fas protein expression in response to TNFα treatment. Actin serves as loading control.

Supplementary Figure 2: TNFα-induced Fas mRNA and protein expression are impaired in p65 knockout or IκBα dominant-negative expressing MEFs. (a) RT-qPCR analysis of Fas mRNA (shown as fold change normalized to L32 mRNA) in Wt 3T3 or SV40 immortalized Wt or p65 knockout (KO) MEFs after 4 and 8 h of TNFα treatment. **(b)** FACS analysis of surface Fas expression in Wt and p65 KO SV40 MEFs after 14 h of TNFα treatment. Both the induction of Fas mRNA and protein surface expression by TNFα are abrogated when p65 expression is lost. Fas protein surface expression even seemed to be controlled by p65 in the absence of TNFα treatment. **(c)** Caspase-3/-7 activity of total lysates of SV40 MEFs transiently transfected with empty pcDNA3.1. vector or pcDNA3.1. containing a cDNA encoding for a phosphorylation-deficient, dominant negative mutant of IκBα (IκBDN), either untreated, treated with TNFα or FasL alone or pretreated with TNFα for 14 h before adding FasL for 4 h. IκBαDN expression abolishes the TNFα-induced sensitization to FasL-induced caspase-3 activation. a.u.: arbitrary units. Values shown represent the mean ± SD of three independent experiments (a and b). In (c) the horizontal line represents the mean of three independent experiments. **(d)** Anti-IκBα and anti-Fas western blot analysis of total

lysates of SV40 MEFs expressing empty pcDNA3.1 or IκBαDN, either untreated, treated with TNFα or FasL alone or pretreated with TNFα for 14 h before adding FasL for 3 or 6 h. Actin serves as loading control. Overexpression of IκBαDN diminishes Fas expression and TNFα-induced Fas upregulation.

Supplementary Figure 3: TNFα induces NFκB activation and increased Fas protein expression in primary mouse hepatocytes and Hepa1-6 cells. Anti-phospho IκBα, anti-IκBα, anti-phospho p65 **(a)** or anti-Fas **(b)** western blots analysis of total extracts of primary hepatocytes treated with TNFα for different time points. Actin or tubulin serve as loading controls. TNFα triggers early IκBα phosphorylation and subsequent degradation as well as concomitant p65 phosphorylation. After 1 h the levels of IκBα were restored. Fas expression started to increase after 4-6 h of TNFα treatment. **(c)** The same analysis as in (a) but with Hepa1-6 cells.

Supplementary Figure 4: Validation of *de novo* SplashRNA predictions for the knockdown of Fas and p65 in Hepa1-6 cells. (a) Schematic map of the validated constitutive (pMSCV) retroviral miR-E expression vector and western blot analysis of several miRE-shFas predictions. The six miRE-shFas predictions (Fas1-6) and a miRE-shRenilla were cloned into the indicated retroviral vector and then expressed in 3T3 MEFs at single-copy conditions to determine the knockdown efficiency of each single oligonucleotide. Knockdown efficiencies are depicted and β-actin serves as loading control. **(b)** Anti-Fas western blot analysis of total extracts of Hepa1-6 expressing the Fas-1 shRNA. Knockdown efficiency is ca. 70%. Tubulin serves as loading control. **(c)** Caspase-3/-7 activity in total lysates of shRenilla or shFas Hepa1-6 cells, either untreated, treated with TNFα or FasL alone or pretreated with TNFα for 14 h before adding FasL for 4 h. Knockdown of Fas expression significantly decreases FasL-induced caspase-3 activation. Values shown are the means of three independent experiments ± SD. **(d)** Anti-p65, anti-Fas, anti-IκBα and anti-Bcl-x_L western blot analysis of total extracts of shRenilla and shp65 and Wt Hepa1-6 cells showing an efficient (70%) knockdown of p65 expression concomitant with diminished expression of the NFκB target genes IκBα and Bcl-x_L. Tubulin serves as loading control.

Supplementary Figure 5: p65 and Fas surface expression in the liver of PBS or TNFα-treated mice injected with shp65 plasmids via the tail vein. Mice were hydrodynamically injected through the tail vein with transposon vectors carrying p65 shRNAs plus a vector carrying a transposase. After 5 days, they were i.p injected either with PBS (left panels) or TNFα (right panels) and sacrificed after 14 h. Liver slices were incubated with anti-mFas, anti-p65, anti-GFP or IgG isotype control (data not shown) primary antibodies followed by incubation with anti-rabbit-Alexa 594 (red, p65 or Fas) and anti-chicken-Alexa 488 (green, GFP) secondary antibodies. **(a)** Upper panel shows GFP+ cells with p65 red staining, lower panel shows only p65 red staining of both, PBS and TNFα treated mice. p65 expression is clearly diminished in GFP+ cells due to shp65 expression (compare upper panel and lower panel, see arrows). **(b)** Fas expression (red staining) is increased on the surface of hepatocytes (arrows) after injecting the mice with TNFα but Fas content in GFP+ shp65 expressing hepatocytes is difficult to assess (see text).
