# Supplementary figures and images for: TNFα sensitizes hepatocytes to FasL-induced apoptosis by NFκB-mediated Fas upregulation
Source: Cell Death Dis. 2018 Sep 5;9(9):909. doi: 10.1038/s41419-018-0935-9 (PMC6125596; doi:10.1038/s41419-018-0935-9)

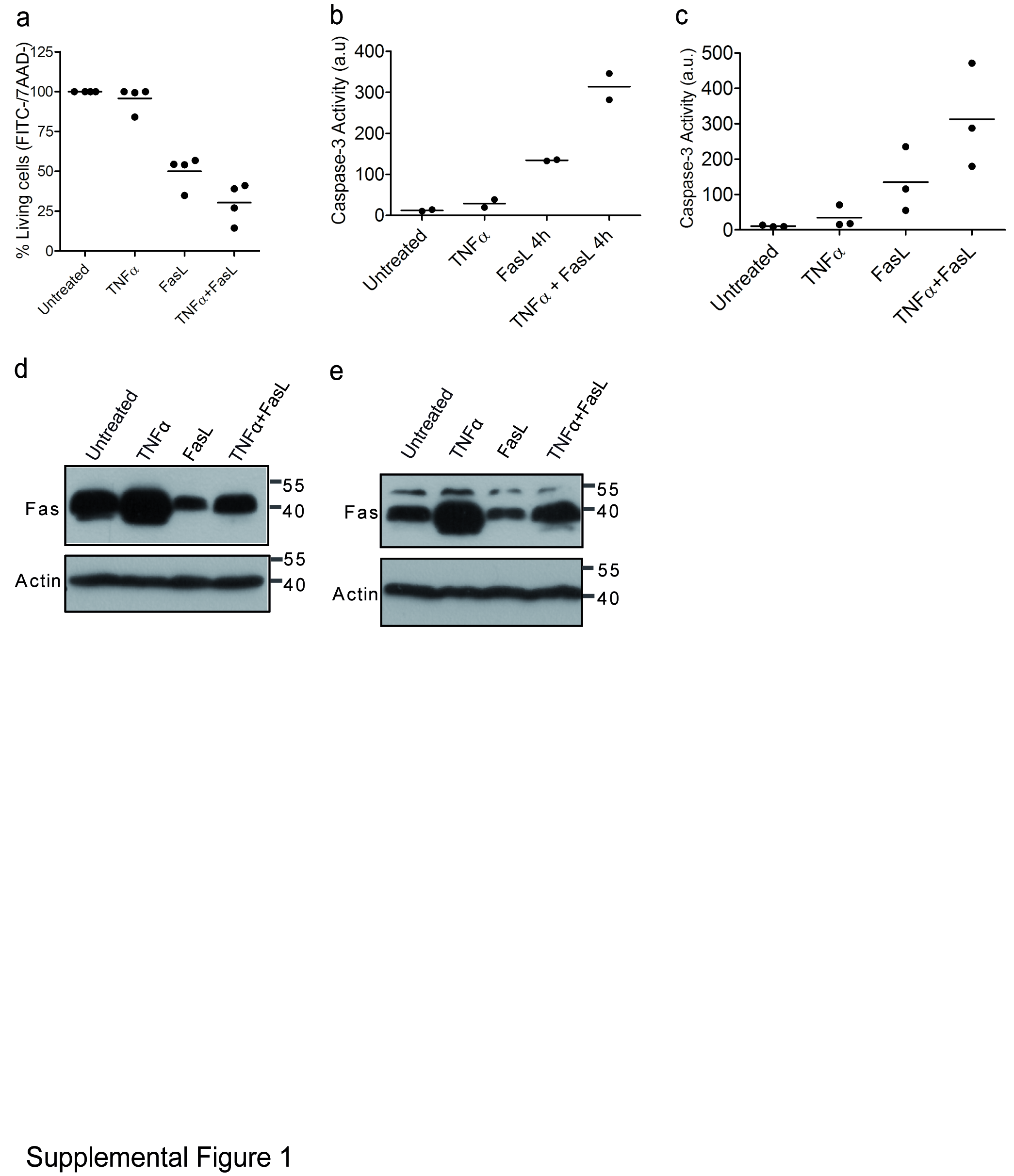

Supplement: Supplementary file 2 — Figure S1 [file 41419_2018_935_MOESM2_ESM.tif]

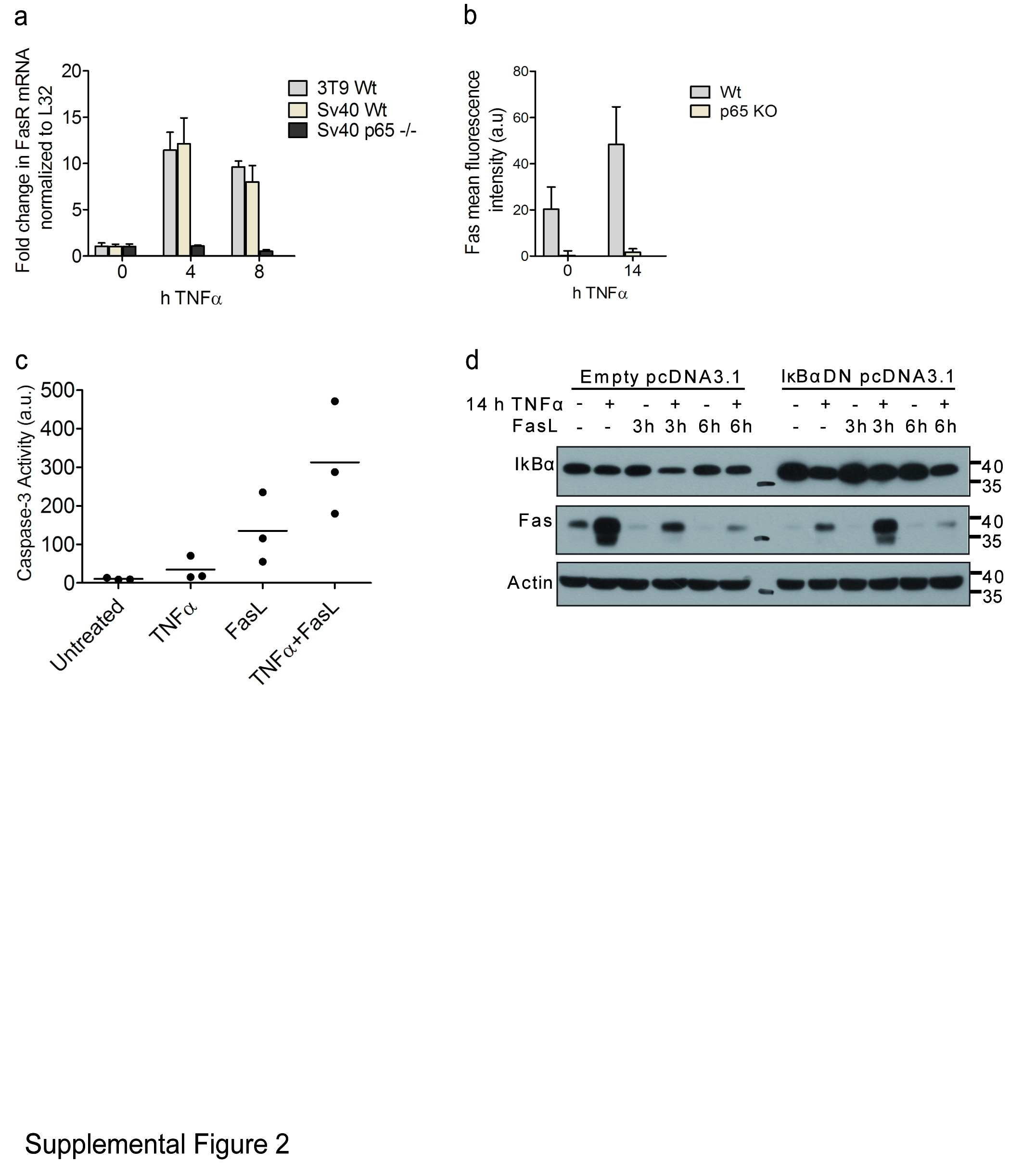

Supplement: Supplementary file 3 — Figure S2 [file 41419_2018_935_MOESM3_ESM.tif]

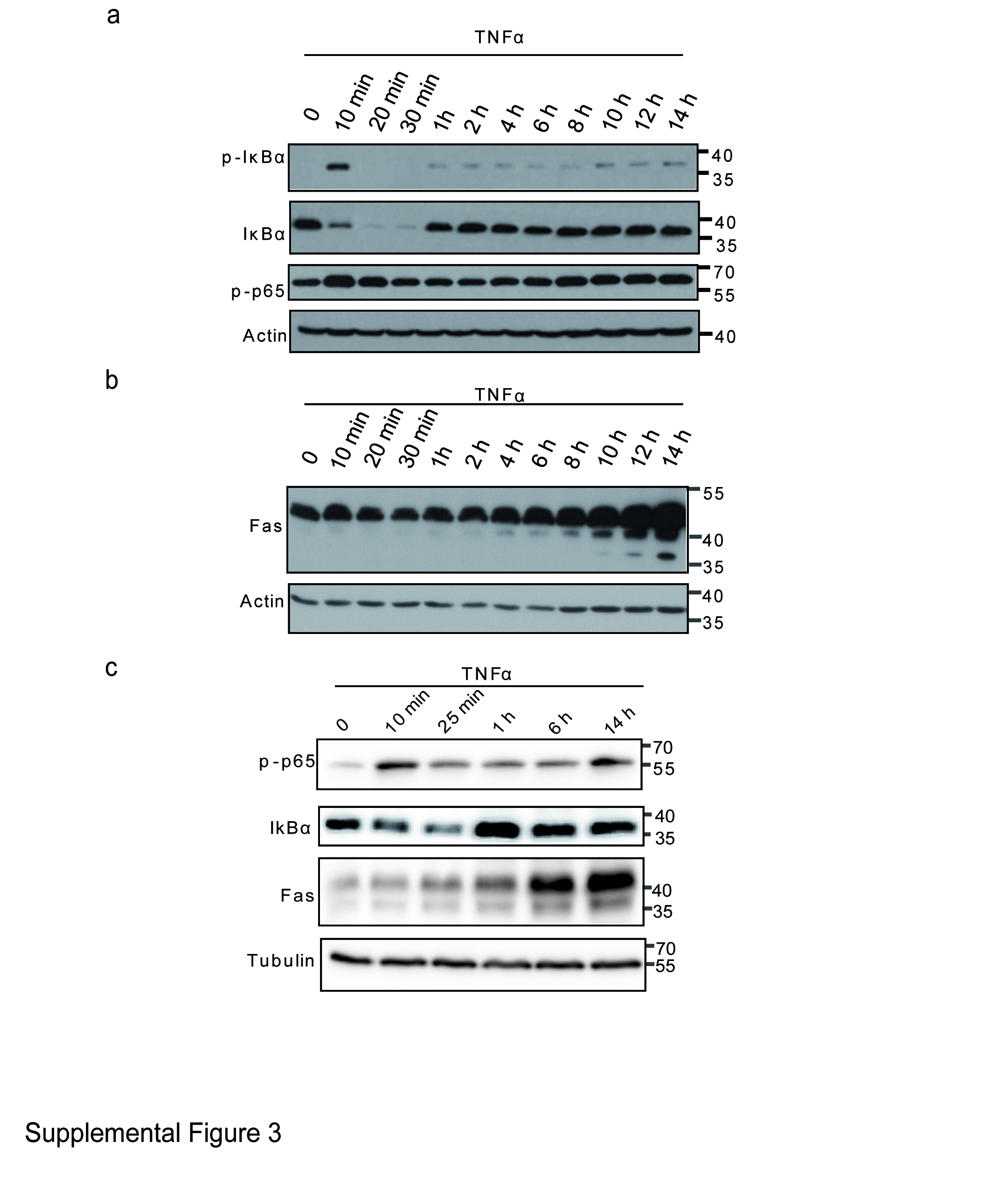

Supplement: Supplementary file 4 — Figure S3 [file 41419_2018_935_MOESM4_ESM.tif]

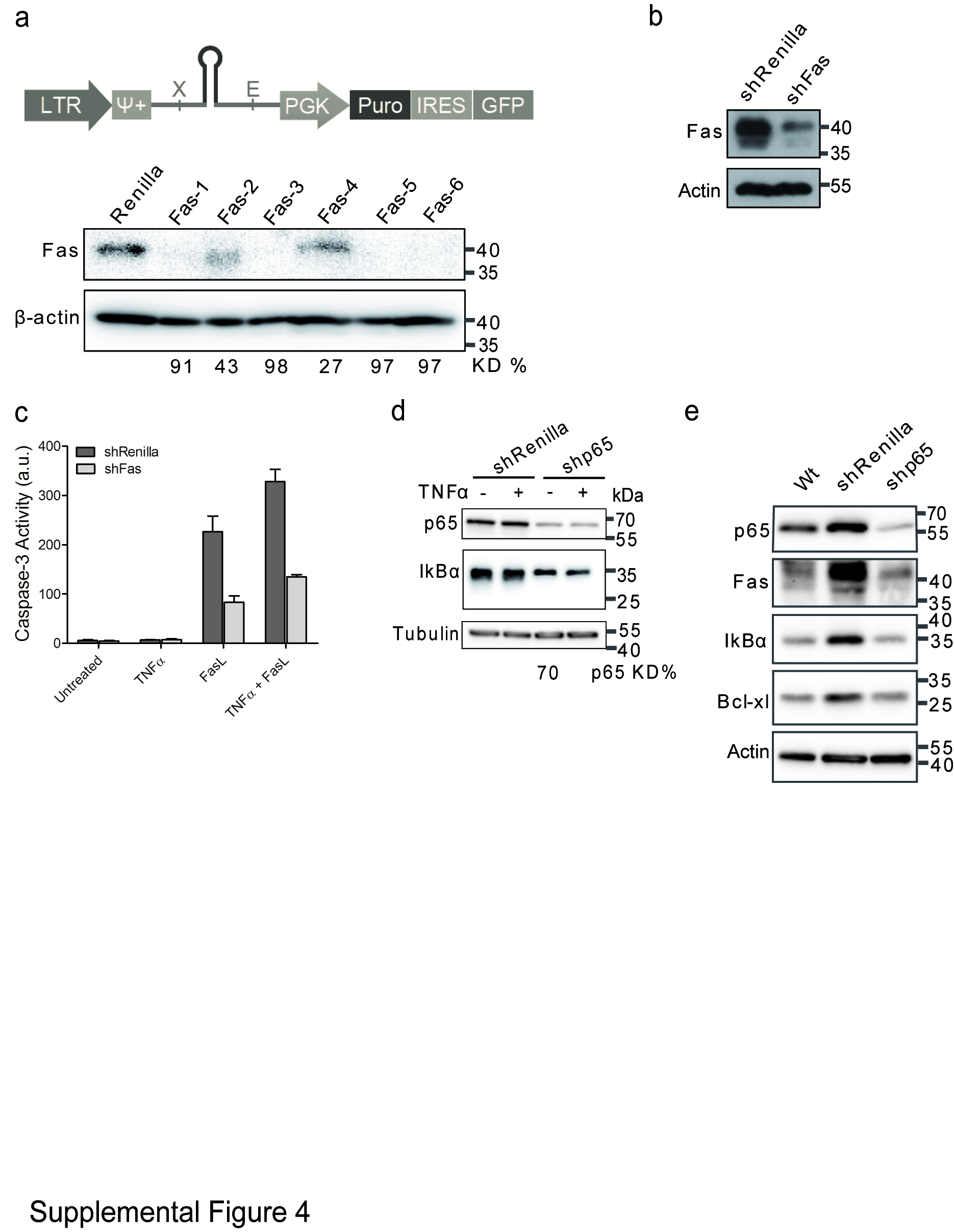

Supplement: Supplementary file 5 — Figure S4 [file 41419_2018_935_MOESM5_ESM.tif]

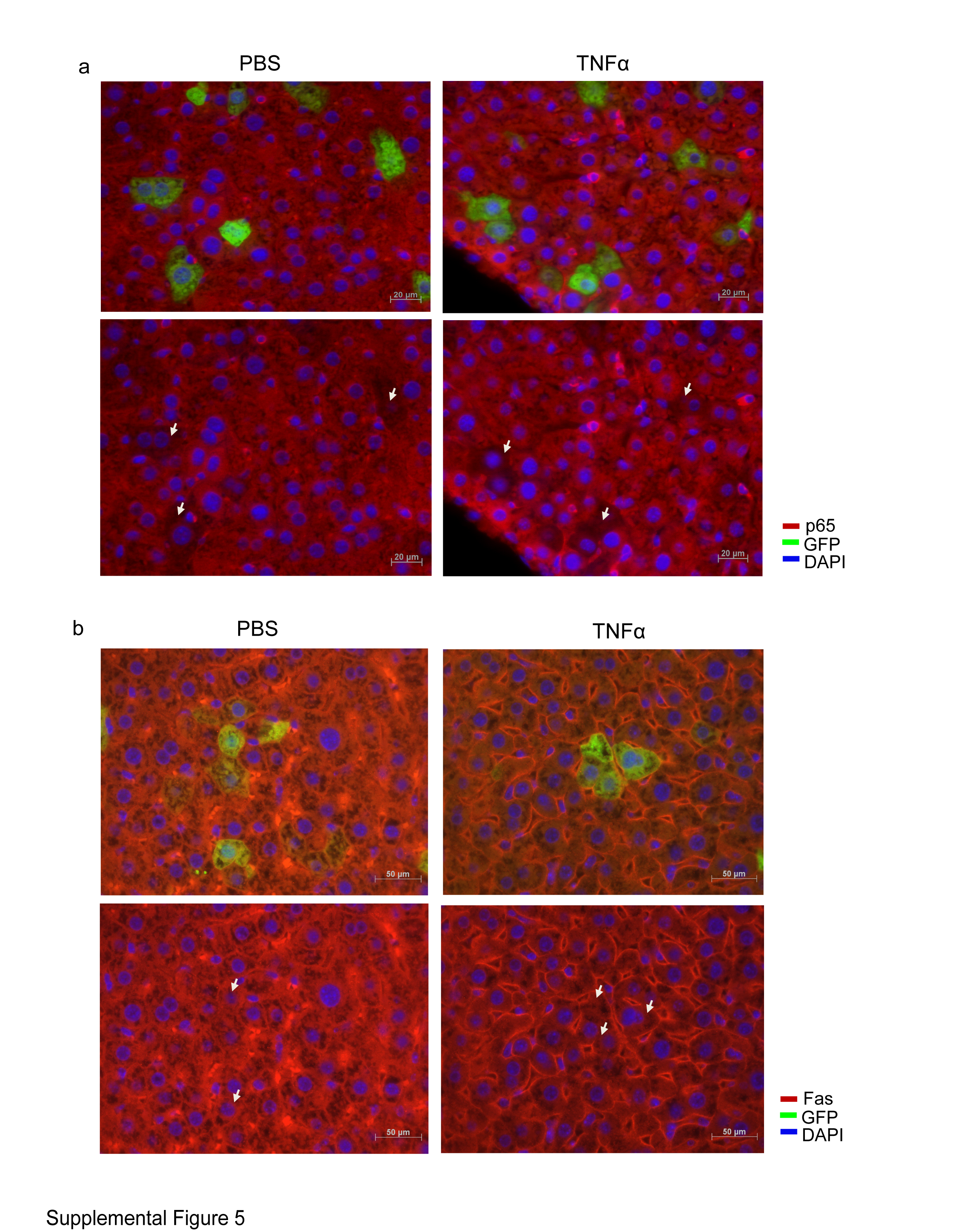

Supplement: Supplementary file 6 — Figure S5 [file 41419_2018_935_MOESM6_ESM.tif]
