# Supplementary material for: TNFα sensitizes hepatocytes to FasL-induced apoptosis by NFκB-mediated Fas upregulation
Source: Cell Death Dis. 2018 Sep 5;9(9):909. doi: 10.1038/s41419-018-0935-9 (PMC6125596; doi:10.1038/s41419-018-0935-9)

Original – Scans of Westernblots

Figure 2

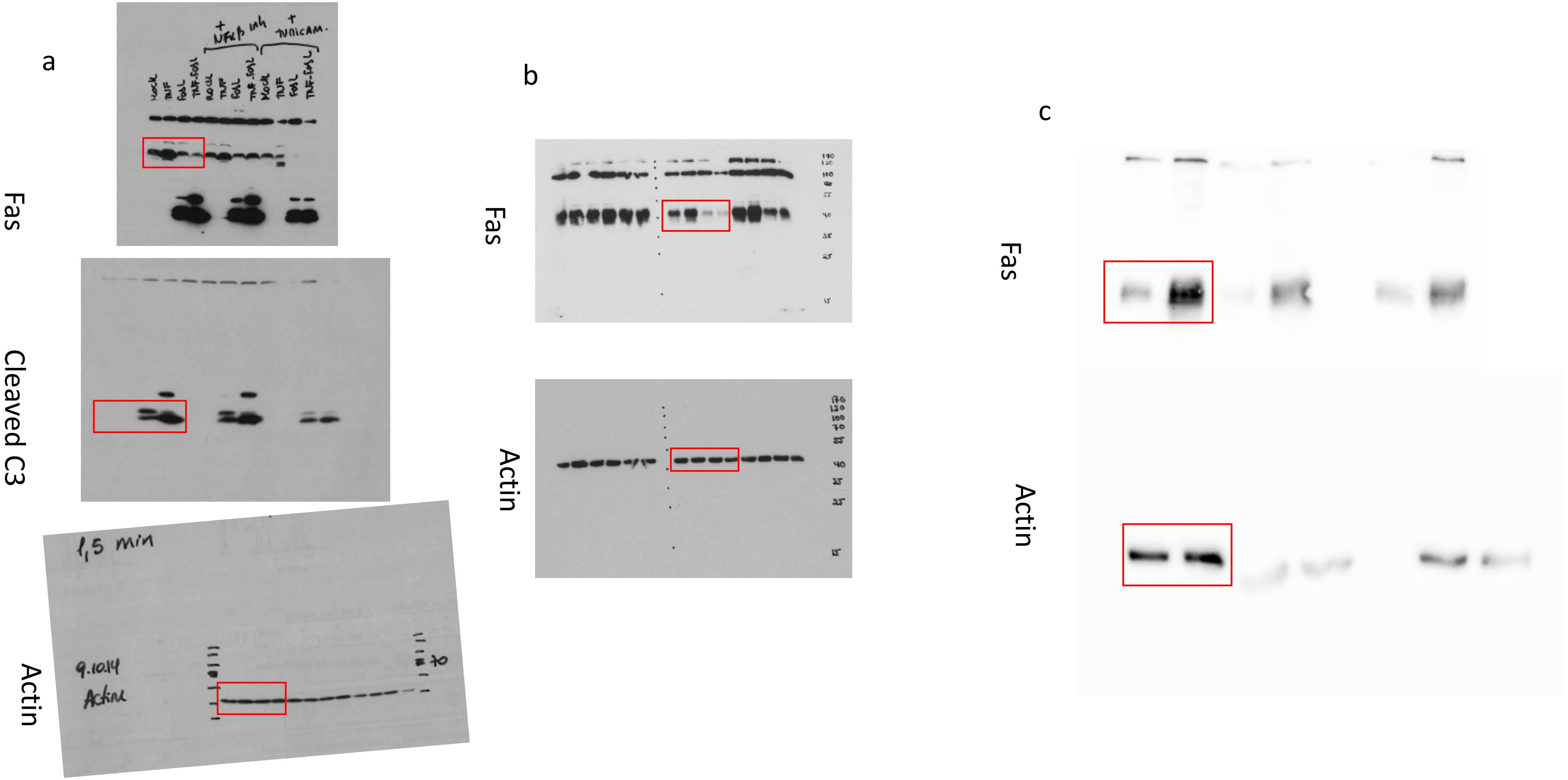

Original – Scans of Westernblots
Figure 3

a

Fas

Tubulin

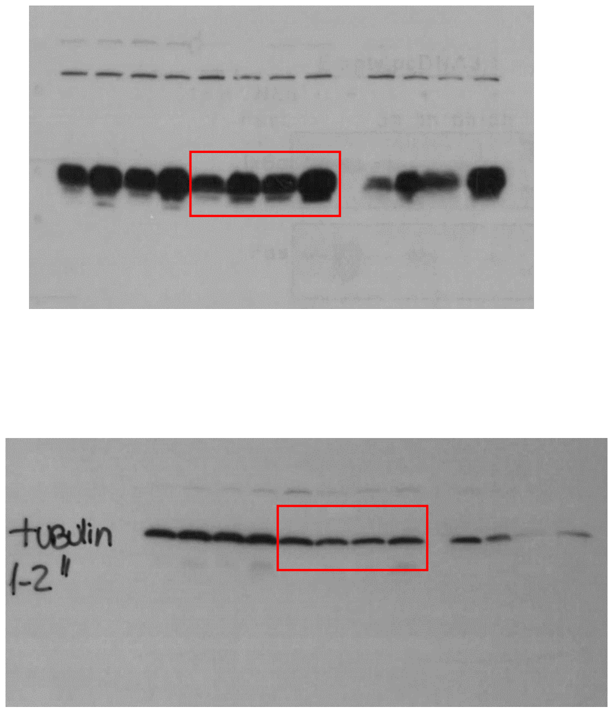

b

Fas

E-Cadherin

Tubulin

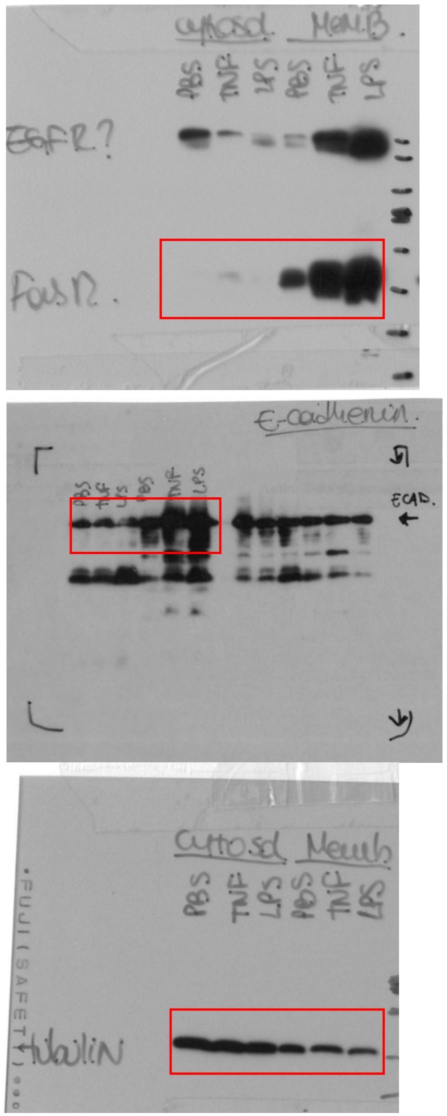

Original – Scans of Westernblots

Figure 4

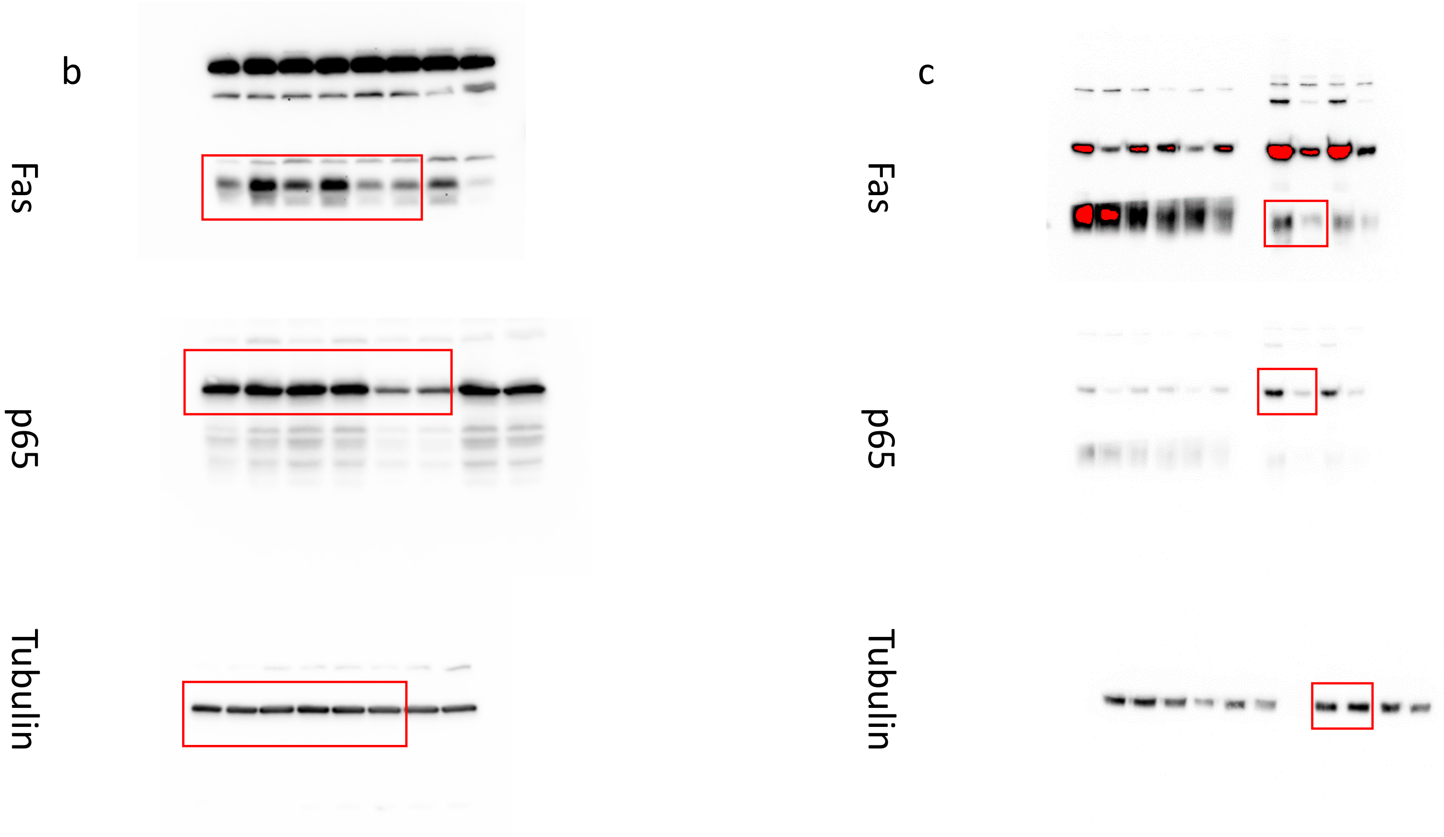

Original – Scans of Westernblots

Figure 5

c

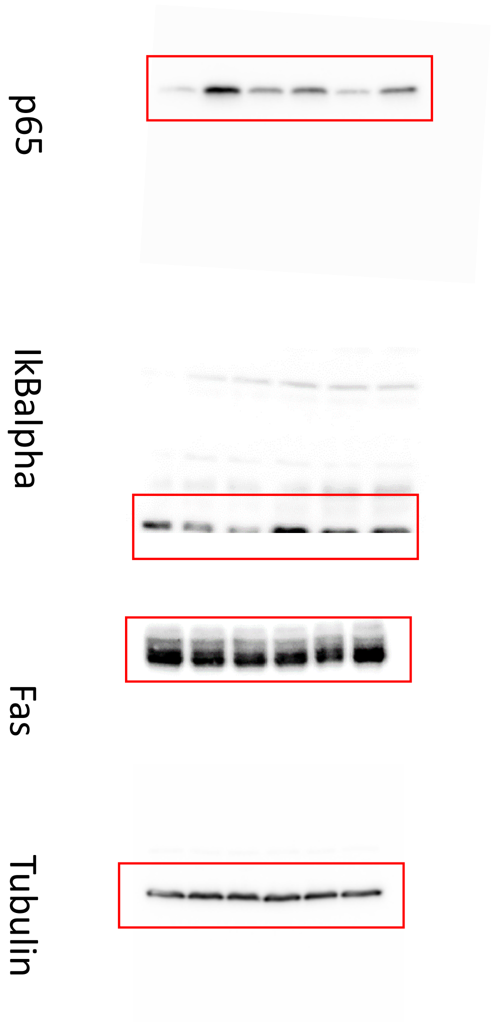

d

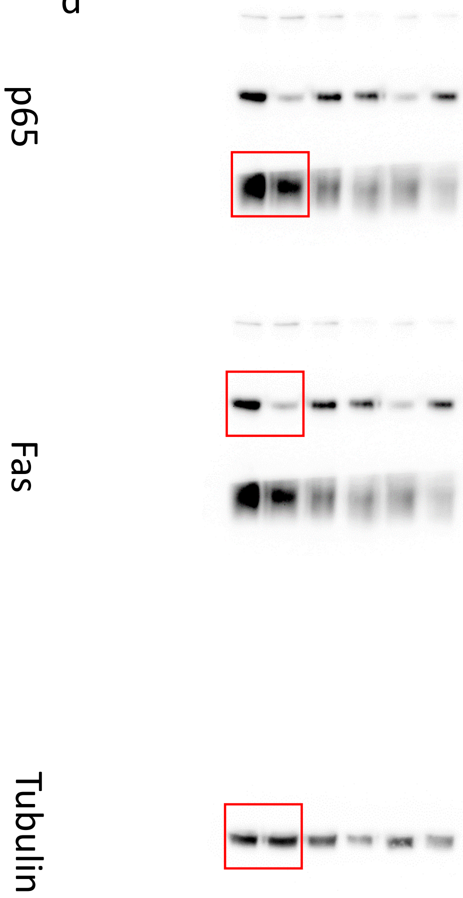

Original – Scans of Westernblots

Supplementary figure 1

d

Fas

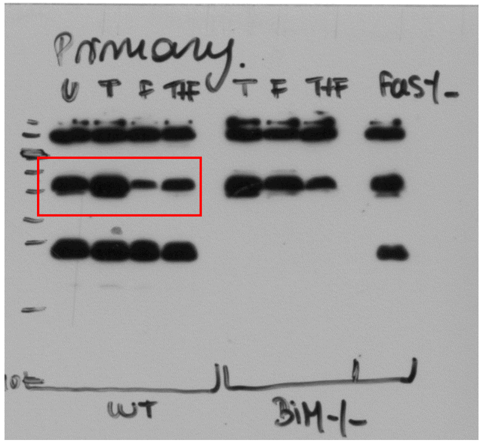

Actin

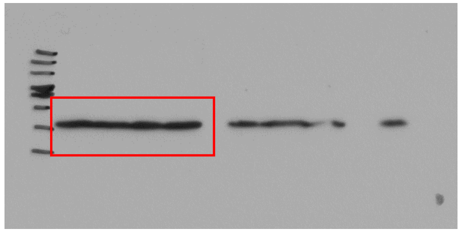

e

Fas

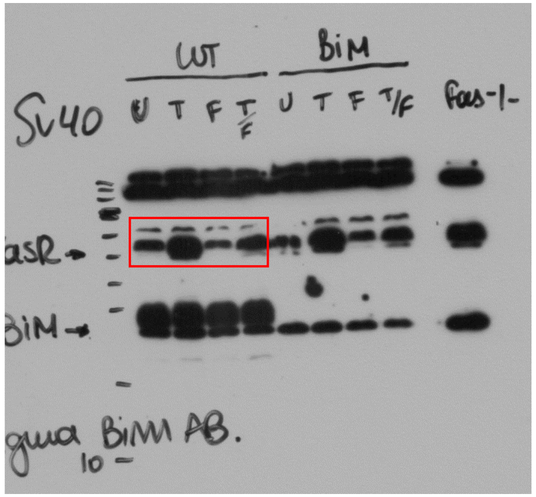

Actin

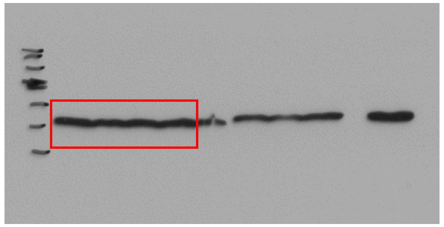

Original – Scans of Westernblots

Supplementary figure 2

d

Fas

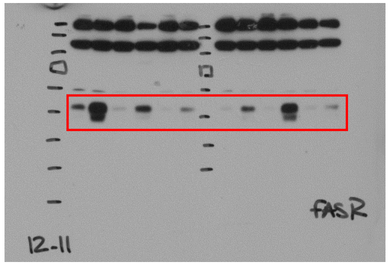

IkB

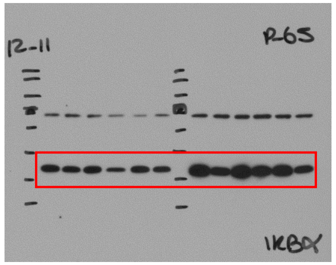

Actin

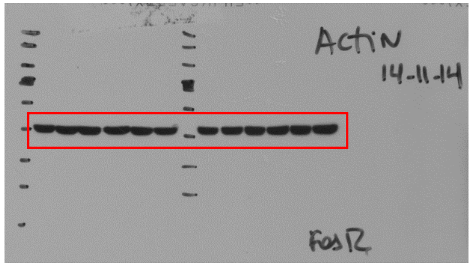

Original – Scans of Westernblots
Supplementary figure 3

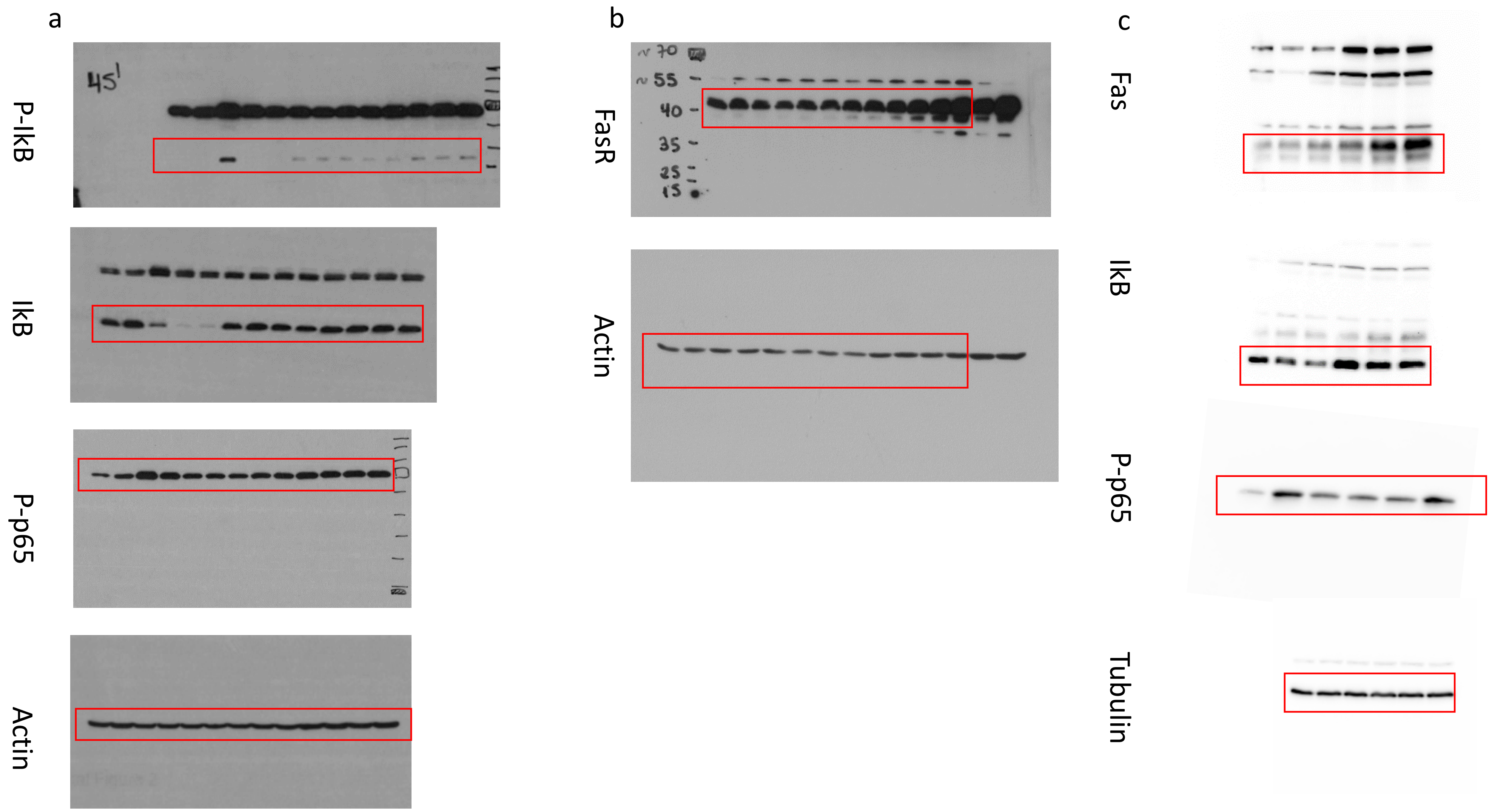

Original – Scans of Westernblots
Supplementary figure 4

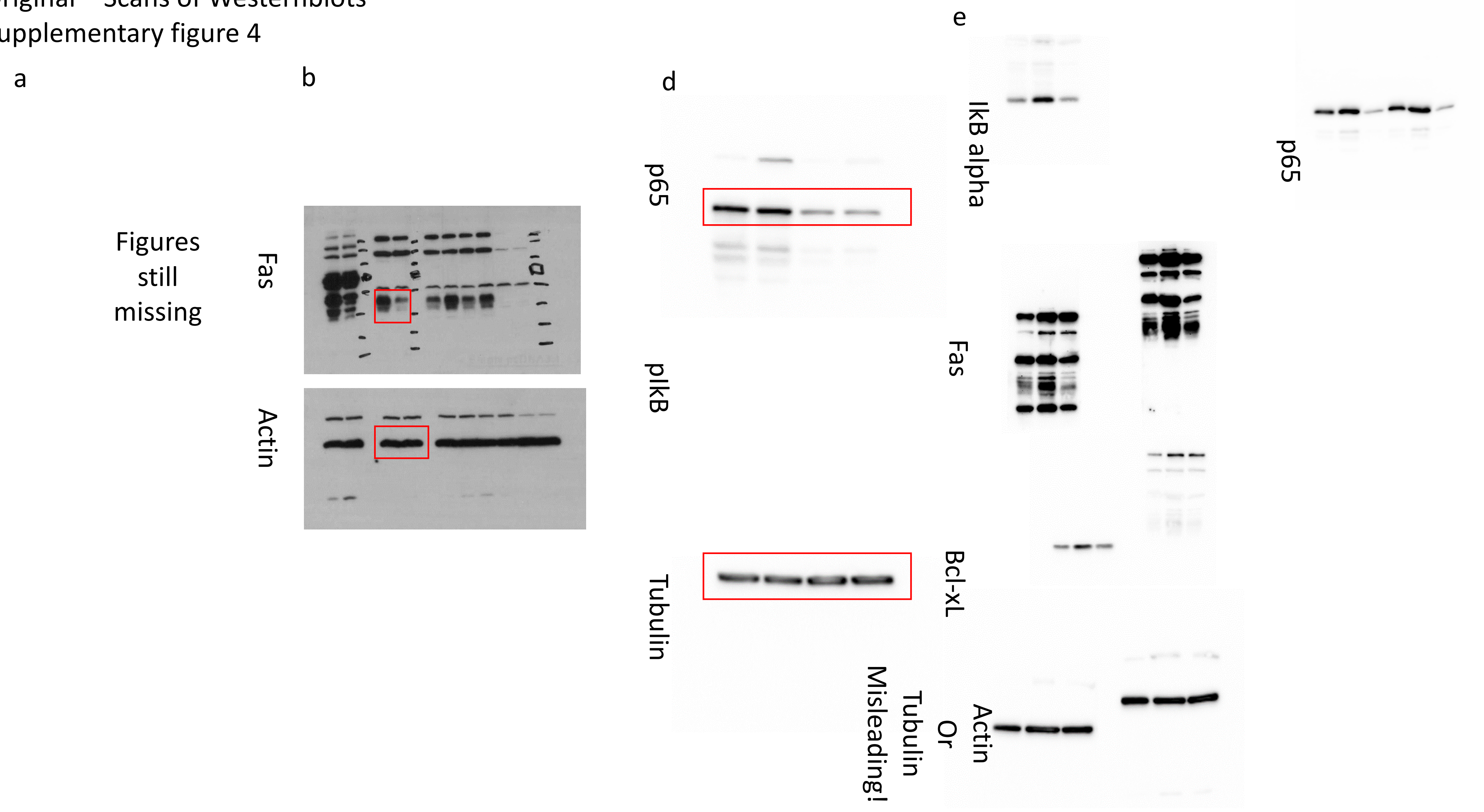

Supplement: Supplementary file 7 — Figure S6 [file 41419_2018_935_MOESM7_ESM.pdf]
